# Leveraging Function Intersectionality and Multi-Modal Cerebrovascular Reactivity Measures for the Derivation of Individualized Intracranial Pressure Thresholds in Acute Traumatic Neural Injury

**DOI:** 10.3390/bioengineering12050485

**Published:** 2025-05-02

**Authors:** Kevin Y. Stein, Logan Froese, Alwyn Gomez, Amanjyot Singh Sainbhi, Nuray Vakitbilir, Abrar Islam, Tobias Bergmann, Frederick A. Zeiler

**Affiliations:** 1 Biomedical Engineering, Price Faculty of Engineering, University of Manitoba, Winnipeg, MB R3T 5V6, Canada; amanjyot.s.sainbhi@gmail.com (A.S.S.); vakitbir@myumanitoba.ca (N.V.); islama9@myumanitoba.ca (A.I.); bergmant@myumanitoba.ca (T.B.); frederick.zeiler@umanitoba.ca (F.A.Z.); 2 Max Rady College of Medicine, Rady Faculty of Health Sciences, University of Manitoba, Winnipeg, MB R3E 3P5, Canada; 3 Department of Clinical Neuroscience, Karolinska Institutet, 171 77 Stockholm, Sweden; log.froese@gmail.com; 4 Section of Neurosurgery, Department of Surgery, Rady Faculty of Health Sciences, University of Manitoba, Winnipeg, MB R3E 3P5, Canada; gomeza35@myumanitoba.ca; 5 Division of Anaesthesia, Department of Medicine, Addenbrooke’s Hospital, University of Cambridge, Cambridge CB2 0QQ, UK; 6 Pan Am Clinic Foundation, Winnipeg, MB R3M 3E4, Canada

**Keywords:** personalized physiologic targets, intracranial pressure, individualized intracranial pressure thresholds, traumatic brain injury

## Abstract

It has been proposed that subject-specific intracranial pressure (ICP) thresholds can be feasibly derived using the relationship between cerebrovascular reactivity and ICP. Such individualized intracranial pressure (iICP) thresholds have been suggested to have more robust associations with long-term outcomes of post-traumatic brain injury (TBI) than current guideline-based thresholds. However, both existing works have derived iICP using solely the pressure reactivity index (PRx) and a threshold of +0.20. Therefore, the goal of this study was to validate prior works and compare various cerebrovascular reactivity indices for their utility in deriving iICP. A custom iICP derivation algorithm was developed. Then, using existing archived human datasets from the Winnipeg Acute TBI Database, iICP thresholds were derived using three cerebrovascular reactivity indices: PRx, the pulse amplitude index (PAx), and the RAC (correlation (R) between the pulse amplitude of ICP (A) and cerebral perfusion pressure (C)). The yield of iICP derivation was found to vary significantly, depending on the cerebrovascular reactivity index and threshold used. A logistic regression analysis was then used to evaluate and compare the abilities of each index-derived iICP to predict the 6-month outcome. Among all index–threshold combinations tested, only PRx > 0 was able to produce an iICP that was able to outperform guideline-based ICP thresholds. PRx-based iICP seems to be superior to both PAx- and RAC-based iICP for predicting long-term outcomes. However, further work is needed to identify the ideal cerebrovascular reactivity thresholds for iICP derivation.

## 1. Introduction

The detrimental effects of acute biomechanical insult to the central nervous system, also termed traumatic brain injury (TBI), are a result of both primary brain injury, the initial structural damage to the brain occurring at the time of the incident, and secondary brain injury, the ongoing damage resulting from downstream derangements in cerebral physiology [1]. While there is little that can be done to reverse primary injury, the mechanisms involved in secondary brain injury are potential targets for therapeutic intervention [2]. Therefore, the main focus of moderate to severe TBI management is to prevent such secondary injury mechanisms, in particular intracranial hypertension and cerebral ischemia. This is accomplished by therapeutically maintaining intracranial pressure (ICP) and cerebral perfusion pressure (CPP) within their physiologic ranges. Current management guidelines recommend an ICP threshold of 20 mmHg or 22 mmHg and a CPP target range of 60–70 mmHg [3,4]. However, despite significant improvements in our ability to achieve these targets in recent decades, the poor outcomes associated with moderate to severe TBI have remained relatively unchanged [1,5,6].

It has been suggested that this limited improvement in patient outcomes is in part due to a lack of consideration for individual phenotypes, with many studies having demonstrated significant heterogeneity in cerebral physiologic response to TBI [7,8,9,10]. For instance, studies by Czosnyka et al. in 2005 and 2008 revealed age and sex-related outcome differences, with older patients and certain female sub-cohorts experiencing higher mortality rates [11,12]. Genetic factors have also been shown to play a role, with certain polymorphisms, such as in the apolipoprotein E gene, being associated with worse outcomes [13]. Using various physiologic, clinical, and demographic factors, Åkerlund et al. were even able to identify six distinct TBI endotypes that provided prognostic utility [14]. Furthermore, recent studies have revealed that a significant portion of cerebral physiologic insult burden in TBI does not respond to guideline-based treatments and that their effects differ between various patient subgroups [15,16,17,18,19].

This high degree of variability in treatment response has exposed the significant limitations of traditional one-size-fits-all treatment paradigms. Therefore, there is an increasing interest in the development of personalized medicine approaches to TBI management. One such approach is the concept of cerebral perfusion pressure optimum (CPPopt), which was first introduced in 2002 by Steiner et al. [20]. The authors were able to demonstrate that a U-shaped relationship exists between CPP and cerebrovascular reactivity and that it is feasible to use this relationship to identify a personalized CPP target that optimizes one’s cerebral autoregulatory capacity. When this personalized target was compared to guideline-based CPP targets, it was revealed to have superior associations with long-term outcomes [21].

Following the reasoning behind CPPopt, a personalized ICP threshold should also be possible to derive using the relationship between ICP and cerebrovascular reactivity. However, in spite of the promising findings in regard to CPPopt, exploration of personalized ICP thresholds has been incredibly limited, with only two studies published on the topic so far [22,23]. In the original 2014 article that first proposed individualized ICP (iICP) thresholds, Lazaridis et al. plotted ICP against the pressure reactivity index (PRx; correlation between ICP and mean arterial pressure [MAP]) and, through manual inspection, identified the ICP value past which cerebrovascular reactivity became persistently deranged [22]. Using a PRx threshold of +0.20 to represent the transition point between intact and impaired cerebrovascular reactivity, the authors were able to identify an iICP threshold in approximately 68% of patients. They found that ICP doses above these identified iICP values were stronger predictors of mortality than an ICP dose above guideline-based thresholds.

The second work done on iICP thresholds was performed by Zeiler et al. in 2021 [23]. The authors were able to develop a semi-autonomous algorithm for the methodology laid out in the original study, leveraging the intersection between two defined functions. This algorithm had an accuracy of approximately 83.2%. The authors then corrected for any errors that the algorithm produced. Using a multi-centered cohort, iICP thresholds were identifiable in 65.3% of patients, falling well in line with the original study. Upon univariate logistic regression analysis, the authors found that mean hourly doses with ICP above iICP thresholds produced a stronger association with mortality compared to ICP doses above 20 or 22 mmHg.

Though the preliminary findings discussed above are quite promising, the methods for deriving iICP require the entire recording period, hindering clinical applicability. Therefore, a continuous method of calculating iICP, similar to those developed for CPPopt [24,25,26], is required. However, before such work can begin, a more accurate algorithm for iICP identification is needed. Further, both existing works have only used PRx for iICP identification. This is problematic, as the pulse amplitude of ICP (AMP)-based indices, such as the pulse amplitude index (PAx—the correlation between AMP and MAP) and RAC (the correlation (R) between AMP (A) and CPP (C)), have been suggested to be better predictors of long-term outcome than PRx in some sub-group populations [27,28]. Therefore, here, we attempt to develop an improved iICP identification algorithm, leveraging function intersectionality, and we compare the utility of PRx, PAx, and RAC in iICP calculation and outcome prediction.

## 2. Materials and Methods

### 2.1. Patient Population

This study utilized existing archived data from the Winnipeg Acute TBI Database [29]. This repository includes data from all adult TBI patients (≥18 years of age) who were admitted to the surgical intensive care unit (SICU) of the Health Sciences Centre (Winnipeg, MB, Canada) for invasive ICP and arterial blood pressure (ABP) monitoring. All patients suffered a moderate (Glasgow Coma Score [GCS] 9–12) to severe (GCS of <8) TBI and, in accordance with the Brain Trauma Foundation (BTF) guidelines, received standard-of-care management, including maintaining ICP below 20 or 22 mmHg and CPP above 60 mmHg [3]. It should be noted that local management practices do not aggressively manage elevated CPP under most circumstances. ICP was monitored using intra-parenchymal strain gauge probes (Codman ICP MicroSensor; Codman & Shurtlef Inc., Raynham, MA, USA) placed in the frontal lobe or external ventricular drains (EVD; Medtronic, Minneapolis, MN, USA; *n* = 4), while ABP was monitored using radial arterial lines connected to pressure transducers (Baxter Healthcare Corp. CardioVascular Group, Irvine, CA, USA) zeroed at the level of the tragus [30,31].

### 2.2. Data Collection

As part of the ongoing Winnipeg Acute TBI Database, the following data were prospectively collected from patients’ bedside charts: demographic information, admission characteristics, imaging profiles, treatment descriptions, and outcome grading. Additionally, all high-frequency physiologic signals available from patient SICU monitors were recorded in time-series at a frequency of 100 Hz or more using Intensive Care Monitoring “Plus” (ICM+) (Cambridge Enterprise Ltd., Cambridge, UK, http://icmplus.neurosurg.cam.ac.uk). Recordings were initiated within 24 h of each patient’s SICU admission and achieved using either direct digital data transfer or analog-to-digital signal conversion (DT9804/DT9826, Data Translations, Marlboro, MA, USA).

In order to ensure data quality, ICP and ABP signal artifacts were removed in ICM+ by the data collection team using both automated and manual artifact-clearing techniques. Specifically, data segments with implausible values—defined as below 0 mmHg and above 100 mmHg (for ICP) or 300 mmHg (for ABP)—were excluded. Segments lacking waveform morphology (i.e., static signals) were also removed, as they represent various non-physiologic events, such as monitor disconnection, radial line flushing, or patient movement. Drain-opening artifacts were also eliminated in cases where EVDs were used to monitor ICP. Examples of data segments that were identified as artifacts and removed can be found in Appendix A.

Following discharge from the SICU, patients participated in routine follow-up appointments at 1, 3, and 6 months. During these sessions, their overall outcome status was assessed using the Glasgow Outcome Scale-Extended (GOSE) [32]. These evaluations were performed by experienced specialist surgeons through structured interviews with the patients and, if applicable, their designated proxies. Finally, all data were fully anonymized and stored securely. For the purposes of this study, all data collected between January of 2019 and December of 2023 were acquired.

### 2.3. Ethics

Data collection for the Winnipeg Acute TBI Database was approved by the University of Manitoba Health Research Ethics Board (H2017:181, H2017:188), the Shared Health Services Manitoba Research Impact Committee, and the Patient Privacy Offices of Manitoba (RI2017:078 and RI:2017:076). Since all data were thoroughly anonymized, to the extent that they cannot be traced back to any individual patients, both the research ethics board and the provincial Patient Privacy Offices of Manitoba granted approval for data collection to occur under a waived-consent model. Approval for retrospectively accessing the database for analysis purposes was also granted by the local ethics board (H2020:118, B2023:001, H2024:217, and H2024:266).

### 2.4. Signal Processing

Post-acquisition signal processing was performed using ICM+. To derive AMP, Fourier analysis was conducted on the ICP pulse waveform for each 10-second window of data [27]. Next, a 10-second non-overlapping moving average filter was employed to down-sample ICP and ABP (resulting in MAP) in order to focus on the frequency range relevant to cerebrovascular reactivity [33,34] and to minimize the effects of the respiratory cycle [5]. CPP was then calculated by subtracting ICP from MAP: CPP = MAP–ICP. To assess cerebrovascular reactivity, three ICP-based indices were derived: PRx, PAx, and RAC. PRx was derived by calculating the Pearson correlation between 30 consecutive 10-second windows of ICP and MAP, updating every minute [35,36]. PAx and RAC were calculated in a similar manner using AMP and MAP, and AMP and CPP, respectively [27,37]. Finally, all data were down-sampled to a minute-by-minute resolution and subsequently exported as comma-separated value files for further processing in R Statistical Computing Software (Version 4.1.0, R Core Team (2020). R: A language and environment for statistical computing. R Foundation for Statistical Computing, Vienna, Austria. URL: https://www.R-project.org/).

### 2.5. iICP Derivation

Working off the methods outlined in the previous two studies [22,23], a custom automated algorithm was created using R Statistical Computing Software to calculate iICP using the relationship between cerebrovascular reactivity and ICP. In order to compare the utility of various ICP-based cerebrovascular reactivity indices, iICP was derived using three indices: PRx, PAx, and RAC. Thresholds were chosen by referencing recent literature that outlines thresholds that are best able to discriminate between outcomes: 0, +0.25, and +0.35 were chosen for PRx; 0, +0.20, and +0.25 were chosen for PAx; and 0 was chosen for RAC [38,39,40]. This produced seven index–threshold pairs for iICP derivation.

**Figure 1:**
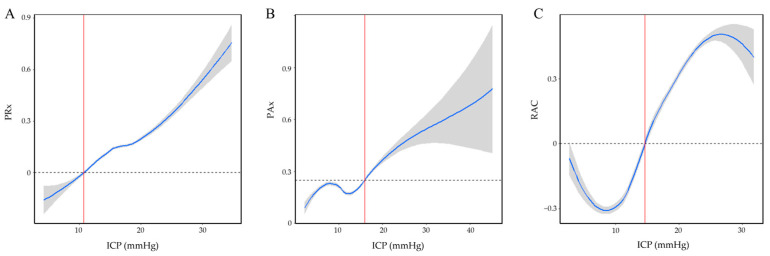
Patient examples of LOESS curves with 95% confidence intervals used for the determination of iICP. (**A**) iICP derivation using PRx > 0. (**B**) iICP derivation using PAx > 0.25. (**C**) iICP derivation using RAC > 0. Each example was sourced from a different patient. Dashed lines indicate the cerebrovascular reactivity threshold used. Red lines demarcate the identified iICP value. ICP = intracranial pressure, PAx = pulse amplitude index, PRx = pressure reactivity index, RAC = correlation between slow waves of AMP and CPP.

For each patient and index–threshold pair, our custom-built algorithm performed the following to identify iICP. First, a locally weighted scatterplot smoothing (LOESS) model was fit between ICP and the cerebrovascular reactivity index of interest (i.e., PRx, PAx, or RAC) using the patient’s entire recording period. Values for the cerebrovascular reactivity index were then estimated for every 0.01 mmHg increment of the dataset’s ICP range, with 95% confidence intervals calculated through bootstrapping. Next, the model was plotted, and the first ICP value at which the index surpassed the chosen threshold (moving from below threshold to above) was identified as the iICP, as long as the index remained above the threshold for at least 10 mmHg of ICP following this value. This was done to prevent the algorithm from identifying points where the curve only transiently crossed the threshold. In cases where there was less than 10 mmHg of data following the identified point available, such as in cases where the identified point is near the upper end of the data range, the index was only required to remain above the threshold for the length of the data that were available. The algorithm did tolerate dips if up to 0.01 a.u. below the cerebrovascular reactivity threshold without disqualifying the identified point. Patient examples of LOESS curves for iICP determination are presented in Figure 1.

To ensure the accuracy of the algorithm-derived thresholds, each LOESS function plot was manually inspected. Next, the algorithm-derived thresholds and manually identified thresholds were compared, with any discrepancies between the two being corrected for prior to analysis. Lastly, two filtered versions of iICP were also determined. The first included only iICP values that had confidence intervals of less than 0.2 a.u. in the LOESS plot, which was designated as iICP.ci. This was done to help comment on whether limiting confidence intervals would help improve the prognostic utility of iICP. The second variation included only iICP values between 5 mmHg and 25 mmHg, designated as iICP.5-25. This was done under the consideration that, if iICP were to become clinically implemented at some point, values outside of this range would likely be ignored, defaulting to guideline-based thresholds.

### 2.6. Data Processing

Prior to statistical analysis, final data processing was performed using R statistical computing software. Mean values of all physiologic variables were calculated for each patient, as well as % times above/below thresholds. Next, mean hourly doses of ICP above guideline-based thresholds (20 mmHg and 22 mmHg), as well as the various identified iICP values, were computed for each patient. The mean hourly dose was calculated using the methodology outlined by Zeiler et al. [23]. In short, all minute-by-minute data points at which ICP was greater than the threshold of interest were first identified. Then, for all the identified data points, the following computation was performed: dose = ICP–ICP threshold. All doses across the entire recording period were then summated and divided by the total recording duration (in hours), generating a mean hourly dose. We opted to use this metric over % time, as the mean hourly dose takes both duration and magnitude into consideration, thus offering a more detailed description of the cerebral insult burden than % time.

### 2.7. Statistical Analysis

A statistical analysis was performed using R statistical computing software with the following add-on packages: *MASS*, *purrr*, *fmsb*, *pROC*, *broom*, *verification*, *tidyverse*, *ggplot2*, *dplyr*, and *zoo.* All continuous variables were assessed for normality using the Shapiro–Wilk test. All physiologic variables were found to be non-parametric and were consequently summarized using medians and interquartile ranges (IQRs). Demographic data were summarized using raw counts or, where appropriate, medians and IQRs. Percentage yields of iICP calculations for each derivation method were calculated. Histograms were then created for each index–threshold pair to illustrate the distributions of iICP values for the entire patient cohort.

Next, patients were dichotomized based on 6-month GOSE scores into *Alive* (GOSE 2–8) versus *Dead* (GOSE 1) and *Favorable* (GOSE 5–8) versus *Unfavorable* (GOSE 1–4). Mann–Whitney U and Chi-square testing were then used to evaluate for any differences in continuous and non-continuous variables, respectively, between the dichotomized groupings. Box plots were then created to illustrate differences in mean hourly doses of ICP above iICP thresholds between the outcome groupings. Additionally, for each index–threshold pairing, patients were dichotomized based on whether an iICP was *Identified* vs. *Not Identified*, with Mann–Whitney U and Chi-square testing once again used to assess for differences between the groups. This was done to compare patient datasets in which an iICP threshold was or was not identifiable and to identify factors contributing to its presence.

Univariate logistic regression analysis was then used to evaluate the association between the various iICP derivations and 6-month outcomes. Bootstrapping techniques were used to compute the area under the curve (AUC) and its associated confidence interval (CI) for each model. Akaike information criteria (AIC), *p*-values, and Nagelkerke’s pseudo-R^2^ values were also calculated. Receiver operating characteristic (ROC) curves were then created to visually illustrate the performance of each iICP method for predicting outcomes. For all iICP derivations that produced a greater AUC than the guideline-based thresholds, the DeLong test was performed to assess whether the difference in AUCs was statistically significant. Finally, multivariable logistic regression analysis was employed to confirm that associations found would remain significant after adjusting for admission characteristics with known associations with long-term outcomes. As one of the most recognized clinical prognostication tools used in TBI management, the International Mission for Prognosis and Analysis of Clinical Trials (IMPACT) Core model, which includes age, admission GCS motor score, and pupillary response (normal bilaterally, unilaterally unreactive, or bilaterally unreactive), was used [41,42]. For all statistical testing, an alpha value of 0.05 was set for significance. Given the exploratory nature of this study, *p*-values did not undergo corrections for multiple comparisons.

## 3. Results

### 3.1. Patient Population

A total of 124 patients from the Winnipeg Acute TBI Database were included in this study. Four of these patients had their ICP monitored using an EVD, while intra-parenchymal strain gauge probes were used for the rest of the cohort. The median age of the cohort was 42 years of age (IQR: 27–57), with 83% of the cohort being males. Median admission GCS was 6.5 (IQR: 4–8). At 6 months post-TBI, 62% of the cohort was still alive (GOSE 2–8) and 59% had a favorable outcome (GOSE 5–8). A more comprehensive summary of patient demographics can be found in Appendix B. The median duration of physiology recording was 73.3 h (IQR: 37.2–130.3).

### 3.2. Derivation of iICP

The median % times with ICP above 20 mmHg and 22 mmHg were 1.48% (IQR: 0.08–5.75%) and 0.78% (0–2.97%), respectively, indicating that ICP was generally well controlled in the cohort. Median % times with ICP above iICP thresholds varied among the seven index–threshold pairs used for derivation, from as low as 0.33% (0.10–1.53%) for PAx > 0.25 to as high as 19.05% (3.11–74.89%) for PRx > 0. A more detailed cerebral physiologic summary can be found in Appendix C.

The percentage yields for the various iICP derivation methods can be found in Table 1. PRx > 0.35 produced the greatest yield among the index–threshold pairs, while RAC > 0 produced the lowest. PRx > 0.25 produced a yield of 69.35%, which falls well in line with the 65.3% and 68% yields produced through the previous two studies, both of which used PRx > 0.20 to derive iICP [22,23]. Both iICP.ci and iICP.5-25 achieved lower yields than iICP for all index–threshold pairs; however, the extent to which they were lower varied drastically.

**Figure 2:**
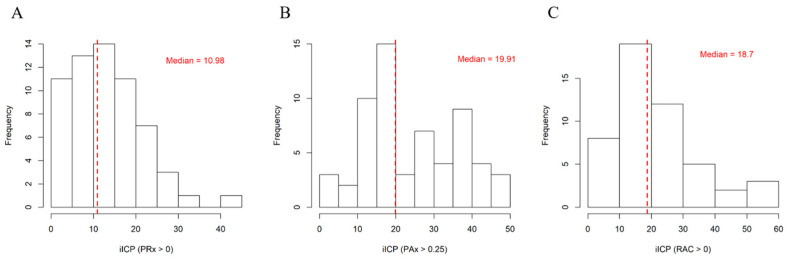
Histograms showing the distributions of iICP calculations for the entire cohort. (**A**) iICP derived using PRx > 0. (**B**) iICP derived using PAx > 0.25. (**C**) iICP derived using RAC > 0. Red dashed lines demarcate median values. iICP = individualized intracranial pressure threshold, PAx = pulse amplitude index, PRx = pressure reactivity index, RAC = correlation (R) between slow waves of AMP (A) and CPP (C).

Histograms illustrating the distributions of iICPs calculated for the patient cohort can be found in Figure 2 (PRx > 0, PAx > 0.25, RAC > 0) and Appendix D (PRx > 0.25, PRx > 0.35, PAx > 0, PAx > 0.20). Derivations using lower thresholds tended to have lower median iICP values. iICP derived using PRx > 0 had the smallest median value, at 10.98, while PAx > 0.25 had the greatest, at 19.91.

Upon the manual inspection of each LOESS curve produced during iICP derivation (124 patients × 7 index–threshold pairs = 868 plots total), it was determined that our custom algorithm was able to correctly identify iICP in 99.19% of cases. The algorithm incorrectly identified an iICP value when one did not exist in two cases, failed to identify an iICP when one did exist in three cases, and identified the wrong value as the iICP in two cases.

### 3.3. Comparisons Between Dichotomized Groupings

**Figure 3:**
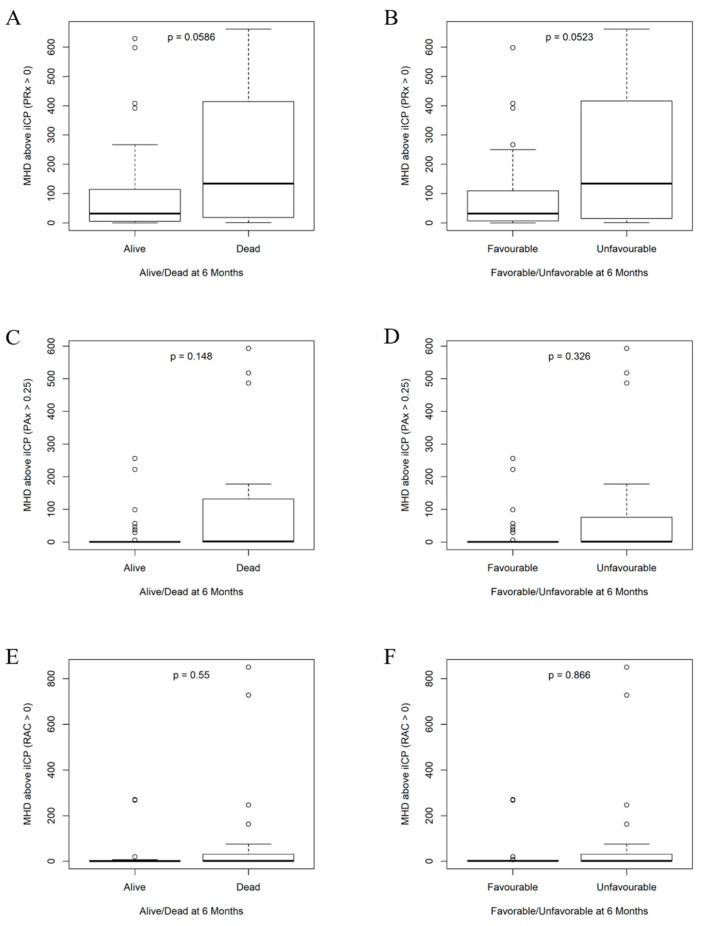
Box plots of mean hourly doses above iICP thresholds for Alive/Dead and Favorable/Unfavorable at 6 months. (**A**) Mean hourly dose of ICP above iICP (PRx > 0) for Alive/Dead. (**B**) Mean hourly dose of ICP above iICP (PRx > 0) for Favorable/Unfavorable. (**C**) Mean hourly dose of ICP above iICP (PAx > 0.25) for Alive/Dead. (**D**) Mean hourly dose of ICP above iICP (PAx > 0.25) for Favorable/Unfavorable. (**E**) Mean hourly dose of ICP above iICP (RAC > 0) for Alive/Dead. (**F**) Mean hourly dose of ICP above iICP (RAC > 0) for Favorable/Unfavorable. Alive (GOSE 2–8), Dead (GOSE 1), Favorable (GOSE 5–8), Unfavorable (GOSE 1–4). *p*-values were calculated using the Mann–Whitney U test. iICP = individualized intracranial pressure threshold, MHD = mean hourly dose, PAx = pulse amplitude index, PRx = pressure reactivity index, RAC = correlation (R) between slow waves of AMP (A) and CPP (C).

The results of the Mann–Whitney U and Chi-square tests comparing *Alive* versus *Dead* and *Favorable* versus *Unfavorable* can be found in Table 2. The mean hourly dose of ICP above 20 mmHg was statistically greater in the *Dead* and *Unfavorable* groups. Mean hourly doses of ICP above iICP thresholds failed to reach statistical significance for all seven index–threshold pairs. However, iICP derived using PRx > 0 did demonstrate a sizable difference in means between the outcome groupings and nearly reached statistical significance with *p*-values of 0.0586 and 0.0523 for *Alive* versus *Dead* and *Favorable* versus *Unfavorable*, respectively. Derivation using PAx > 0.25 was the second closest to reaching statistical significance with *p*-values of 0.146 and 0.326. All other pairs produced *p*-values greater than 0.50. Boxplots illustrating the spread of the mean hourly doses of ICP above iICP thresholds can be found in Appendix E. Boxplots for iICP derived using PRx > 0, PAx > 0.25, and RAC > 0 can be found in Figure 3, as these were the best-performing thresholds for each index.

The results of the Mann–Whitney U and Chi-square tests comparing those who had an iICP *Identified* versus *Not Identified* can be found for each index–threshold pair in Appendix F, Appendix G, Appendix H, Appendix I, Appendix J, Appendix K and Appendix L. For iICP derived using PRx > 0 and PAx > 0, cerebrovascular reactivity was found to be worse, in terms of both mean values and % time spent above thresholds, in the *Not Identified* group. For iICP derived using PRx > 0.25, only PRx was found to be worse, while, for iICP derived using RAC > 0, only PAx and RAC were found to be worse. For all other index–threshold pairs, no differences in cerebrovascular reactivity were found. In addition to worse cerebrovascular reactivity, the *Not Identified* group for PAx > 0 derived iICP was found to have a lower mean ICP.

### 3.4. Logistic Regression Analyses

**Figure 4:**
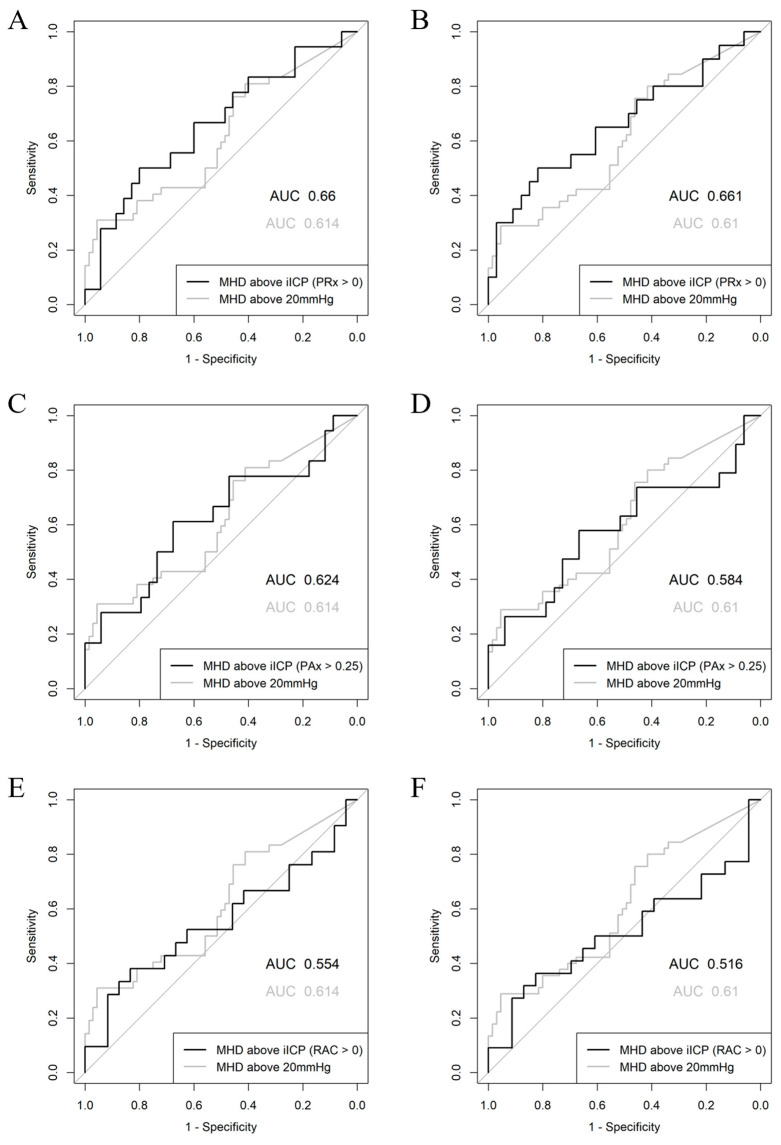
Univariate logistic regression ROC curves for mean hourly doses above iICP thresholds. (**A**) Mean hourly dose of ICP above iICP (PRx > 0) for Alive/Dead. (**B**) Mean hourly dose of ICP above iICP (PRx > 0) for Favorable/Unfavorable. (**C**) Mean hourly dose of ICP above iICP (PAx > 0.25) for Alive/Dead. (**D**) Mean hourly dose of ICP above iICP (PAx > 0.25) for Favorable/Unfavorable. (**E**) Mean hourly dose of ICP above iICP (RAC > 0) for Alive/Dead. (**F**) Mean hourly dose of ICP above iICP (RAC > 0) for Favorable/Unfavorable. Alive (GOSE 2–8), Dead (GOSE 1), Favorable (GOSE 5–8), Unfavorable (GOSE 1–4). AUC = area under the curve, iICP = individualized intracranial pressure threshold, MHD = mean hourly dose, PAx = pulse amplitude index, PRx = pressure reactivity index, RAC = correlation (R) between slow waves of AMP (A) and CPP (C), ROC = receiver operating characteristic.

The results of the univariate logistic regression analysis can be found in Table 3. Mean hourly doses of ICP above the guideline-based thresholds reached statistical significance for both *Alive* versus *Dead*, and *Favorable* versus *Unfavorable*. Among the iICP thresholds, only those derived using PRx > 0 were able to reach significance. This index–threshold pair was able to outperform the guideline-based thresholds with regard to both AUC and AIC. However, the DeLong test did not reveal a statistically significant difference between the AUC of iICP, derived using PRx > 0, and that of the guideline-based thresholds for both Alive vs. Dead (*p* = 0.2551 and *p* = 0.2144 for 20 mmHg and 22 mmHg, respectively) and Favorable vs. Unfavorable (*p* = 0.2046 and *p* = 0.2956) prediction. PAx > 0.25 derived iICP performed the second best among the iICP thresholds; however, it failed to reach statistical significance and performed poorer than guideline-based thresholds. ROC curves for iICP thresholds derived using PRx > 0, PAx > 0.25, and RAC > 0 can be found in Figure 4, while ROC curves for the remaining index–threshold pairs can be found in Appendix M. The univariate analysis results for iICP.ci and iICP.5-25 can be found in Appendix N. iICP.ci and iICP.5-25 generally performed better than unfiltered iICP, especially when AIC values were observed.

Upon multivariable logistic regression analysis, all iICP models were able to reach statistical significance; however, only those derived using PRx > 0 were able to consistently provide additional variance in outcome over the IMPACT Core model. iICP derived using this index–threshold pair was also able to outperform the guideline-based thresholds, adding more additional variance in outcome, as seen in the greater Δ Nagelkerke’s R^2^ values. The results of this analysis can be found in Table 4 and Appendix O.

## 4. Discussion

In this study, we were able to validate the existence of iICP thresholds, develop an automated algorithm to identify such thresholds, and compare the utility of PRx, PAx, and RAC-based iICP for predicting long-term outcomes. Through this, we uncovered multiple interesting findings that deserve highlighting. Firstly, it was observed that there are significant differences in the utility of PRx, PAx, and RAC for the determination of iICP. Overall, we found that PRx provides the greatest utility in regard to both derivation yield and ability to predict outcomes. Considering recent studies have proposed that AMP-based indices may be better predictors of long-term outcome than PRx [27,28], it is somewhat surprising to see that PAx and RAC-based iICP performed so poorly. While there is not a clear explanation for the poor performance of Pax-based iICP, the limited utility of RAC-based iICP can possibly be explained by the fact RAC reflects not only cerebrovascular reactivity but also cerebral compliance [37] and thus potentially does not allow for a proper assessment of the relationship between ICP and cerebrovascular reactivity.

Second, it was found that the threshold used for derivation also significantly affects iICP utility. We observed that, among the three thresholds used to derive PRx-based iICP, PRx > 0 performed the best for predicting outcomes and was the sole index–threshold pair to achieve statistical significance. However, this threshold performed worse than both PRx > 0.25 and PRx > 0.35 in terms of the derivation yield. Looking at the results as a whole suggests that, as the PRx threshold used increases, the prognostic ability becomes poorer while the % yield improves. A reversed pattern can be seen for Pax-based iICP, with the greater the threshold used, the better the prognostic ability and the poorer the % yield. This is quite interesting, as it suggests that there is a potential tradeoff between a greater derivation yield and greater prognostic ability. However, considering that PRx > 0 was the only index–threshold pair to achieve statistical significance, we must be cautious when interpreting these findings. These findings also raise questions of whether other cerebrovascular reactivity thresholds that were not tested in our analysis would potentially perform better.

Another noteworthy observation was that the median iICP generated was observed to be positively related to the threshold used. This is understandable since a positive relationship exists between ICP and cerebrovascular reactivity and moving the horizontal line, representing the threshold, upwards would lead to intersections at greater ICP values. Given this, in conjunction with the fact that ICP was very well controlled in our patient cohort (the mean hourly dose of ICP above 20 mmHg was very limited), it may be plausible that a significantly greater amount of data points at which ICP was above iICP were available for the PRx > 0 derivation method. This further complicates our results since this could have contributed to the superior performance of this index–threshold pair in our statistical analysis. However, the fact that Pax-based iICP demonstrated a reversed pattern challenges this concern.

When iICP derived using PRx > 0 is compared with the guideline-based thresholds of 20 mmHg and 22 mmHg, the results suggest that the iICP threshold may potentially offer superior outcome prediction ability. Upon univariate analysis, this PRx > 0 based iICP consistently produced a greater AUC and smaller AIC values for both outcome dichotomizations. Although the DeLong test failed to demonstrate that this difference in AUC was statistically significant, this may have been a result of poor statistical power, considering the reduction in sample size when only selecting patients with an identifiable iICP (39.52–72.58%). Upon multivariable analysis, this iICP provided greater added variance in outcome to the IMPACT Core model, as assessed through Δ Nagelkerke’s R^2^, than either guideline-based threshold. These findings generally support the findings of the previous two works [22,23]. Interestingly, upon Mann–Whitney U testing, the mean hourly dose of ICP above PRx > 0-based iICP failed to reach statistical significance. However, since the % yield for the derivation of this iICP was approximately 50%, the reduced number of patients included in its testing may have hindered statistical power.

Regarding the filtered variations of iICP, it was found that filtering iICP using confidence interval maximums and ICP range could potentially improve prognostic utility. However, it was also found to variably decrease yields. Therefore, further work is needed to determine whether filtering iICP provides an overall benefit to iICP utility. Lastly, it cannot be understated that the concept of iICP is very much still in its infancy and that the findings of this study in no way suggest the implementation of iICP clinically.

## 5. Limitations

Despite the significant findings uncovered in this study, there are important limitations that must be addressed. Firstly, the sample size used (n = 124) was quite small for such a study. This is compounded by the fact that less than perfect % yields further reduced the number of patients available for each individual statistical test. As an example, since the % yield for PRx > 0 based iICP was approximately 50%, only 62 patients were included in its testing. This may have prevented tests from reaching statistical significance in multiple instances. The box plots in Figure 3 hint at this being a possibility, as they all show visual differences in the spread of mean hourly doses despite failing to achieve statistical significance. Future multi-centered work is needed to resolve this issue. Next, our study only tested a handful of thresholds for iICP derivation. This leaves major questions on how other thresholds would perform compared to those evaluated here. Lastly, a major limitation of our results is that our population had their ICPs strictly controlled in accordance with guideline-based thresholds. This strongly biases our findings. Unfortunately, this issue is not feasible to circumvent in human TBI populations.

## 6. Future Directions

Despite the promising findings uncovered here and in the previous two iICP studies, much additional work is needed before this concept can even be remotely considered for clinical use. Firstly, despite the high accuracy of our custom-built algorithm, there are various potential alternative methods that could be used to derive iICP. Work investigating such alternative avenues is needed in order to improve iICP derivation methods. Second, a study comparing a large range of thresholds for each cerebrovascular reactivity index is needed in order to clarify which thresholds are best suited to deriving a prognostically useful iICP.

There is also a need for subpopulation analyses comparing those with lower versus higher identified iICPs. This would help improve our understanding of the effects of ICP burden on individual patients and possible contributors to why some patients may require more extreme ICP control. Additionally, further work is needed in comparing those in which iICP is identifiable with those in which it is not, as this would help us better understand what contributes to the presence of an iICP and potentially help us improve algorithmic yields.

Various hemodynamic parameters, such as pulse pressure, blood pressure variability, and cardiac decoupling, have been shown to influence ICP [43,44,45,46]. As such, these hemodynamic factors are likely to play a significant role in modulating ICP-based cerebrovascular reactivity indices, and, by extension, iICP. To better understand how hemodynamic status impacts iICP, future research should explore the complex relationships between iICP and the multidimensional characteristics of ABP. This should include an evaluation of the autocorrelative structures among these variables, which may reveal dynamic interactions that influence iICP.

Lastly, the fact that current iICP derivation methods use entire recording periods prevents any clinical application. Therefore, an algorithm that allows for the continuously updating derivation of iICP using sliding windows of data is required. Such an algorithm will likely closely mirror principle techniques that have been applied in recent continuously updating CPPopt algorithms [24,25,26].

## 7. Conclusions

The presence of iICP was found to vary heavily, depending on the cerebrovascular reactivity index and threshold used during derivation. Using PRx seems to be superior to both PAx and RAC for deriving iICPs with prognostic utility. Additionally, the threshold chosen seems to play a key role as well, with iICP derived using PRx > 0 performing significantly better than iICP derived using PRx > 0.25 or PRx > 0.35. Among all index-threshold combinations tested, only PRx > 0 was shown to produce an iICP that outperforms guideline-based ICP thresholds at long-term outcome prediction. Further work is required to better understand iICP.

## Figures and Tables

**Table 1:** Percentage yields of iICP derivations.

Index-Threshold Used	% Yield		
	iICP	iICP.ci	iICP.5-25
PRx > 0	49.19	44.35	37.90
PRx > 0.25	69.35	53.23	51.61
PRx > 0.35	72.58	50.00	52.42
PAx > 0	62.10	44.35	45.16
PAx > 0.20	51.61	29.03	29.03
PAx > 0.25	48.39	24.19	25.81
RAC > 0	39.52	20.16	25.00

iICP = individualized intracranial pressure threshold, iICP.ci = individualized intracranial pressure threshold (with CI maximum of 0.2), iICP.5-25 = individualized intracranial pressure threshold (with range limit of 5–25), PAx = pulse amplitude index, PRx = pressure reactivity index, RAC = correlation (R) between slow waves of AMP (A) and CPP (C).

**Table 2:** Mann–Whitney U/chi-square testing for alive/dead and favorable/unfavorable at 6 months.

Variable	Alive/Dead Outcome Groups			Favorable/Unfavorable Outcome Groups		
	Alive Median (IQR)	Dead Median (IQR)	*p*-Value	Favorable Median (IQR)	Unfavorable Median (IQR)	*p*-Value
Age (years)	38.5 (25.8–50)	59.5 (36–67)	**<0.0001**	39 (27–50)	58 (34–67)	**0.0002**
Sex (% Male)	83.80%	81%	0.8990	84.60%	80%	0.7090
Admission GCS Total	7 (5.75–8)	6 (4–8)	0.0528	7 (5–8)	6 (4–8)	0.0743
Admission GCS Motor	5 (3–5)	3.5 (1–5)	**0.0062**	5 (3–5)	4 (1–5)	**0.0165**
Admission GCS Eyes	1 (1–2)	1 (1–2)	0.8170	1 (1–2)	1 (1–2)	0.8730
Admission GCS Verbal	1 (1–2)	1 (1–1.75)	0.6670	1 (1–2)	1 (1–1)	0.4380
Admission Pupil Response (% Bilaterally Reactive)	63.20%	59.50%	0.9240	64.60%	57.80%	0.6970
Marshall CT Grade	4 (3–5)	5 (4–5)	**0.0476**	4 (3–5)	5 (4–5)	**0.0257**
Rotterdam CT Grade	4 (3–5)	5 (4–6)	0.0722	4 (3–5)	5 (4–6)	0.0601
Helsinki CT Score	5 (3.75–9)	7.5 (6–9)	**0.0063**	5 (3–9)	7 (5–9)	**0.0064**
Stockholm CT Score	3.1 (2.4–3.55)	3.45 (2.58–4)	0.1120	3 (2.4–3.5)	3.5 (2.8–4)	**0.0351**
Number with Hypoxia Episode	33.80%	33.30%	1.000	32.30%	35.60%	0.8810
Number with Hypotension Episode	13.20%	7.10%	0.4960	12.30%	8.90%	0.7990
Number with Traumatic SAH	95.60%	97.60%	0.9770	95.40%	97.80%	0.8880
Number with Epidural Hematoma	14.70%	4.80%	0.1900	13.80%	6.70%	0.3810
Admission Hemoglobin	135 (115–148)	128 (114–143)	0.3260	135 (114–148)	129 (119–143)	0.5330
Admission Serum Glucose	8.05 (6.9–10.3)	9.8 (7.2–12.1)	0.1110	8.2 (6.9–10.5)	9.4 (7.18–11.5)	0.2320
Length of Hospital Stay	34 (20–59.2)	8 (4–14)	**<0.0001**	32 (20–54)	9 (4–15)	**<0.0001**
Length of SICU Stay	8.5 (4–15.2)	7 (4–11)	0.3500	8 (4–15)	7 (4–14)	0.8670
Mean MAP (mmHg)	84.3 (80.1–87.1)	84.4 (79–90.2)	0.9680	84.4 (80.1–87.2)	84.1 (79–90.2)	0.9280
Mean ICP (mmHg)	8.92 (5.21–12.2)	10.4 (6.82–16.2)	0.1130	8.55 (4.75–12.1)	10.5 (7.02–16.1)	0.0522
% Time ICP > 20 mmHg	1.09 (0–5.09)	1.7 (0.209–21.3)	0.0564	1.06 (0–5.03)	1.74 (0.208–18.6)	0.0372
% Time ICP > 22 mmHg	0.575 (0–2.52)	0.961 (0.117–11)	0.0385	0.545 (0–2.57)	0.947 (0.113–7.32)	0.0464
Mean Hourly Dose of ICP > 20 mmHg	2.56 (0–10.4)	3.39 (0.738–48.1)	**0.0435**	2.35 (0–10.8)	3.49 (0.634–44.7)	**0.0491**
Mean Hourly Dose of ICP > 22 mmHg	1.12 (0–6.41)	2.07 (0.403–34.9)	**0.0292**	1.21 (0–7.25)	1.82 (0.237–32.1)	0.0583
Mean CPP (mmHg)	74.5 (71.1–80.6)	73.6 (68.7–77.5)	0.1220	75.1 (71.2–80.7)	73.4 (68.9–76.9)	**0.0432**
% Time CPP < 60 mmHg	4.1 (0.932–7.53)	3.37 (0.48–16.4)	0.4240	4.13 (0.494–7.71)	3.54 (0.496–14.5)	0.4270
% Time CPP > 70 mmHg	64 (51.8–83)	61.5 (37.6–77)	0.1630	66.2 (52.3–83.9)	58.9 (37.5–74)	0.0672
Mean PRx	0.136 (0.0299–0.27)	0.202 (0.119–0.37)	**0.0173**	0.152 (0.0372–0.272)	0.192 (0.1–0.339)	0.0911
% Time PRx > 0	63.1 (52–82)	70.7 (64.5–87.4)	**0.0326**	63.9 (52.1–82)	69.6 (60–86.6)	0.1270
% Time PRx > 0.25	39.8 (27.3–57.2)	46.5 (36.5–68.9)	**0.0342**	40.2 (27.8–57.3)	45.8 (32.5–64.1)	0.1680
% Time PRx > 0.35	29.2 (19–42.1)	37.7 (27–57.8)	**0.0222**	30.3 (19.5–44.2)	35.5 (23.5–53.4)	0.1240
Mean PAx	−0.0387 (−0.133–0.0735)	0.0713 (−0.0443–0.31)	**0.0017**	−0.00816 (−0.127–0.0803)	0.051 (−0.094–0.268)	**0.0132**
% Time PAx > 0	46.5 (34.7–60.8)	60.9 (43.8–79.2)	**0.0033**	48.2 (34.8–62.3)	59.2 (39.3–77.6)	**0.0201**
% Time PAx > 0.20	24.7 (15.3–37.4)	39.3 (23.4–64.7)	**0.0016**	27.9 (15.8–37.5)	36.7 (21.8–60.2)	**0.0127**
% Time PAx > 0.25	20.3 (12.4–32.4)	33.8 (19.4–60.1)	**0.0014**	23.1 (12.7–33.7)	31.8 (17.3–56)	**0.0114**
Mean RAC	−0.276 (−0.437–−0.132)	−0.118 (−0.279–0.0974)	**<0.0001**	−0.272 (−0.436–−0.125)	−0.123 (−0.284–0.0614)	**0.0006**
% Time RAC > 0	21.6 (10.6–36.2)	36.7 (23–60.8)	**0.0002**	21.7 (11–36.5)	34.7 (21.1–53.9)	**0.0015**
iICP (PRx > 0)	14.9 (6.77–19.2)	7.01 (5.16–10.9)	0.0639	14.9 (7.6–18.5)	7.01 (4.84–11)	0.0571
% Time ICP > iICP (PRx > 0)	13.5 (3.11–45.6)	58.2 (16.2–87.2)	0.0823	13.5 (3.11–40.5)	58.2 (12.7–87.7)	**0.0458**
Mean Hourly Dose of ICP > iICP (PRx > 0)	31.9 (5.39–115)	134 (19.7–389)	0.0586	31.9 (6.53–110)	134 (16.4–415)	0.0523
iICP (PRx > 0.25)	15.3 (10.7–19.9)	13.9 (9.56–19.2)	0.5810	15.3 (10.6–19.7)	14.6 (9.86–19.6)	0.7860
% Time ICP > iICP (PRx > 0.25)	2.12 (0.424–19.5)	6.21 (0.562–34.6)	0.7010	2.61 (0.452–23)	5.43 (0.497–34.1)	0.9340
Mean Hourly Dose of ICP > iICP (PRx > 0.25)	6.77 (0.921–62)	8.01 (1.42–74.8)	0.6680	8.04 (1.08–63.2)	7.68 (1.27–74.2)	0.8720
iICP (PRx > 0.35)	17.4 (11.3–23.3)	16.5 (11.3–21.6)	0.7220	17.1 (11.2–22.6)	16.8 (11.6–22)	0.9400
% Time ICP > iICP (PRx > 0.35)	1.18 (0.439–6.34)	1.66 (0.393–21.2)	0.7690	1.2 (0.507–6.8)	1.4 (0.333–20.9)	1.0000
Mean Hourly Dose of ICP > iICP (PRx > 0.35)	2.88 (0.756–20.3)	2.03 (0.936–48.8)	0.9680	3.68 (0.826–22.8)	1.98 (0.708–48.1)	0.8360
iICP (PAx > 0)	14.7 (8.27–21.3)	12.1 (9.91–15)	0.9180	14 (8.14–20.9)	12.3 (10.2–16.9)	0.8530
% Time ICP > iICP (PAx > 0)	3.52 (0.49–65.2)	11.4 (1.03–71.1)	0.4790	3.77 (0.543–66.3)	10.3 (0.801–71)	0.7360
Mean Hourly Dose of ICP > iICP (PAx > 0)	14.6 (1.42–121)	20 (0.853–261)	0.6620	16.6 (1.49–121)	19.1 (0.575–260)	0.9440
iICP (PAx > 0.20)	19 (13.4–33.2)	16.2 (14.4–28.6)	0.9020	18.7 (13.3–33.4)	17.9 (14.5–29)	0.9290
% Time ICP > iICP (PAx > 0.20)	0.519 (0.15–4.01)	0.438 (0.144–1.19)	0.7740	0.521 (0.17–4.38)	0.357 (0.14–1.17)	0.5310
Mean Hourly Dose of ICP > iICP (PAx > 0.20)	1.02 (0.363–6.59)	1.11 (0.115–3.24)	0.8250	1.08 (0.392–6.71)	0.927 (0.106–3.11)	0.5530
iICP (PAx > 0.25)	20.7 (15.5–36.3)	16.6 (15.1–27.7)	0.3350	20.4 (15.4–36.8)	16.7 (15.1–30)	0.4740
% Time ICP > iICP (PAx > 0.25)	0.343 (0.114–1.31)	0.466 (0.146–31)	0.4060	0.388 (0.129–1.51)	0.371 (0.131–26.5)	0.7210
Mean Hourly Dose of ICP > iICP (PAx > 0.25)	0.633 (0.263–1.74)	1.46 (0.429–104)	0.1480	0.65 (0.264–1.82)	1.31 (0.256–75.6)	0.3260
iICP (RAC > 0)	18.8 (11.7–27.6)	17.8 (14.2–25.8)	0.7960	18.7 (11.5–24.9)	20.2 (14.2–26.7)	0.5660
% Time ICP > iICP (RAC > 0)	0.615 (0.179–0.846)	1.05 (0.194–24.2)	0.4500	0.627 (0.203–0.973)	0.84 (0.0774–18.8)	0.7790
Mean Hourly Dose of ICP > iICP (RAC > 0)	1.04 (0.21–2.78)	1.85 (0.132–31)	0.5500	1.07 (0.303–2.8)	1.4 (0.0958–25.6)	0.8660

Alive (GOSE 2–8), Dead (GOSE 1), Favorable (GOSE 5–8), Unfavorable (GOSE 1–4). Bolded *p*-values are those reaching statistical significance, *p* < 0.05. CPP = cerebral perfusion pressure, CT = computed tomography, GCS = Glasgow Coma Scale, ICP = intracranial pressure, iICP = individualized intracranial pressure threshold, IQR = interquartile range, MAP = mean arterial pressure, mmHg = millimeters of mercury, PAx = pulse amplitude index, PRx = pressure reactivity index, SAH = subarachnoid hemorrhage, SICU = surgical intensive care unit, RAC = correlation (R) between slow waves of AMP (A) and CPP (C).

**Table 3:** Univariate models of iICP for alive/dead and favorable/unfavorable at 6 months.

**Alive/Dead Outcome Groups**				
**Model**	**AUC (95% CI)**	**AIC**	***p*-Value**	**Nagelkerke’s R^2^**
Mean Hourly Dose of ICP > 20 mmHg	0.614 (0.508–0.716)	138.3	**0.0217**	0.140
Mean Hourly Dose of ICP > 22 mmHg	0.623 (0.513–0.728)	139.4	**0.0146**	0.128
Mean Hourly Dose of ICP > iICP (PRx > 0)	0.660 (0.508–0.813)	68.6	**0.0293**	0.085
Mean Hourly Dose of ICP > iICP (PRx > 0.25)	0.530 (0.391–0.661)	102.8	0.3339	0.002
Mean Hourly Dose of ICP > iICP (PRx > 0.35)	0.503 (0.364–0.631)	108.7	0.4838	0.002
Mean Hourly Dose of ICP > iICP (PAx > 0)	0.533 (0.373–0.677)	89.2	0.3309	0.036
Mean Hourly Dose of ICP > iICP (PAx > 0.20)	0.481 (0.320–0.646)	77.4	0.5940	0.039
Mean Hourly Dose of ICP > iICP (PAx > 0.25)	0.624 (0.457–0.783)	66.2	0.0738	0.123
Mean Hourly Dose of ICP > iICP (RAC > 0)	0.554 (0.373–0.726)	63.7	0.2751	0.071
**Favorable/Unfavorable Outcome Groups**				
**Model**	**AUC (95% CI)**	**AIC**	***p*-Value**	**Nagelkerke’s R^2^**
Mean Hourly Dose of ICP > 20 mmHg	0.610 (0.500–0.719)	142.3	**0.0246**	0.123
Mean Hourly Dose of ICP > 22 mmHg	0.605 (0.490–0.711)	143.4	**0.0291**	0.111
Mean Hourly Dose of ICP > iICP (PRx > 0)	0.661 (0.492–0.808)	68.8	**0.0262**	0.134
Mean Hourly Dose of ICP > iICP (PRx > 0.25)	0.512 (0.353–0.628)	103.5	0.4361	0.001
Mean Hourly Dose of ICP > iICP (PRx > 0.35)	0.486 (0.384–0.642)	109.5	0.5859	0.001
Mean Hourly Dose of ICP > iICP (PAx > 0)	0.506 (0.351–0.665)	90.9	0.4721	0.027
Mean Hourly Dose of ICP > iICP (PAx > 0.20)	0.452 (0.384–0.719)	78.7	0.7291	0.032
Mean Hourly Dose of ICP > iICP (PAx > 0.25)	0.584 (0.403–0.748)	67.9	0.1631	0.109
Mean Hourly Dose of ICP > iICP (RAC > 0)	0.516 (0.338–0.694)	64.2	0.4331	0.062

Alive (GOSE 2–8), Dead (GOSE 1), Favorable (GOSE 5–8), Unfavorable (GOSE 1–4). Bolded *p*-values are those reaching statistical significance, *p* < 0.05. AIC = Akaike information criterion, AUC = area under the curve, CI = confidence interval, ICP = intracranial pressure, iICP = individualized intracranial pressure threshold, mmHg = millimeters of mercury, PAx = pulse amplitude index, PRx = pressure reactivity index, RAC = correlation (R) between slow waves of AMP (A) and CPP (C).

**Table 4:** Multivariable models of iICP for alive/dead and favorable/unfavorable at 6 months.

**Alive/Dead Outcome Groups**					
**Model**	**AUC (95% CI)**	**AIC**	***p*** **-Value**	**Nagelkerke’s R^2^**	**Δ Nagelkerke’s R^2^**
IMPACT Core	0.793 (0.698–0.879)	124.2	**<0.0001**	0.344	-
+Mean Hourly Dose of ICP > 20 mmHg	0.855 (0.773–0.926)	107.9	**<0.0001**	0.500	0.156
+Mean Hourly Dose of ICP > 22 mmHg	0.852 (0.768–0.925)	109.5	**<0.0001**	0.487	0.143
+Mean Hourly Dose of ICP > iICP (PRx > 0)	0.914 (0.817–0.984)	49.3	**<0.0001**	0.607	0.263
+Mean Hourly Dose of ICP > iICP (PRx > 0.25)	0.801 (0.684–0.904)	88.3	**<0.0001**	0.358	0.014
+Mean Hourly Dose of ICP > iICP (PRx > 0.35)	0.788 (0.672–0.883)	93.8	**<0.0001**	0.346	0.002
+Mean Hourly Dose of ICP > iICP (PAx > 0)	0.708 (0.570–0.827)	86.3	**0.0024**	0.237	−0.107
+Mean Hourly Dose of ICP > iICP (PAx > 0.20)	0.698 (0.545–0.827)	77.2	**0.0063**	0.217	−0.127
+Mean Hourly Dose of ICP > iICP (PAx > 0.25)	0.742 (0.585–0.869)	66.8	**0.0019**	0.290	−0.054
+Mean Hourly Dose of ICP > iICP (RAC > 0)	0.756 (0.601–0.893)	64.5	**0.0014**	0.259	−0.085
**Favorable/Unfavorable Outcome Groups**					
**Model**	**AUC (95% CI)**	**AIC**	***p*** **-value**	**Nagelkerke’s R^2^**	**Δ Nagelkerke’s R^2^**
IMPACT Core	0.753 (0.653–0.845)	135.1	**<0.0001**	0.262	-
+Mean Hourly Dose of ICP > 20 mmHg	0.801 (0.706–0.884)	123.1	**<0.0001**	0.392	0.130
+Mean Hourly Dose of ICP > 22 mmHg	0.799 (0.701–0.889)	124.7	**<0.0001**	0.378	0.116
+Mean Hourly Dose of ICP > iICP (PRx > 0)	0.871 (0.752–0.965)	56.6	**<0.0001**	0.523	0.261
+Mean Hourly Dose of ICP > iICP (PRx > 0.25)	0.780 (0.662–0.889)	91.3	**<0.0001**	0.325	0.063
+Mean Hourly Dose of ICP > iICP (PRx > 0.35)	0.770 (0.647–0.872)	96.7	**<0.0001**	0.317	0.055
+Mean Hourly Dose of ICP > iICP (PAx > 0)	0.705 (0.565–0.824)	89.1	**0.0024**	0.208	−0.054
+Mean Hourly Dose of ICP > iICP (PAx > 0.20)	0.684 (0.542–0.814)	80.0	**0.0097**	0.179	−0.083
+Mean Hourly Dose of ICP > iICP (PAx > 0.25)	0.726 (0.574–0.858)	69.8	**0.0033**	0.250	−0.012
+Mean Hourly Dose of ICP > iICP (RAC > 0)	0.739 (0.575–0.874)	65.4	**0.0027**	0.241	−0.021

IMPACT Core model consists of age, admission Glasgow Coma Scale–motor score, and admission pupillary response. Alive (GOSE 2–8), Dead (GOSE 1), Favorable (GOSE 5–8), Unfavorable (GOSE 1–4). Bolded *p*-values are those reaching statistical significance, *p* < 0.05. AIC = Akaike information criterion, AUC = area under the curve, CI = confidence interval, ICP = intracranial pressure, iICP = individualized intracranial pressure threshold, IMPACT = International Mission for Prognosis and Analysis of Clinical Trials, mmHg = millimeters of mercury, PAx = pulse amplitude index, PRx = pressure reactivity index, RAC = correlation (R) between slow waves of AMP (A) and CPP (C).

**Figure A1:**
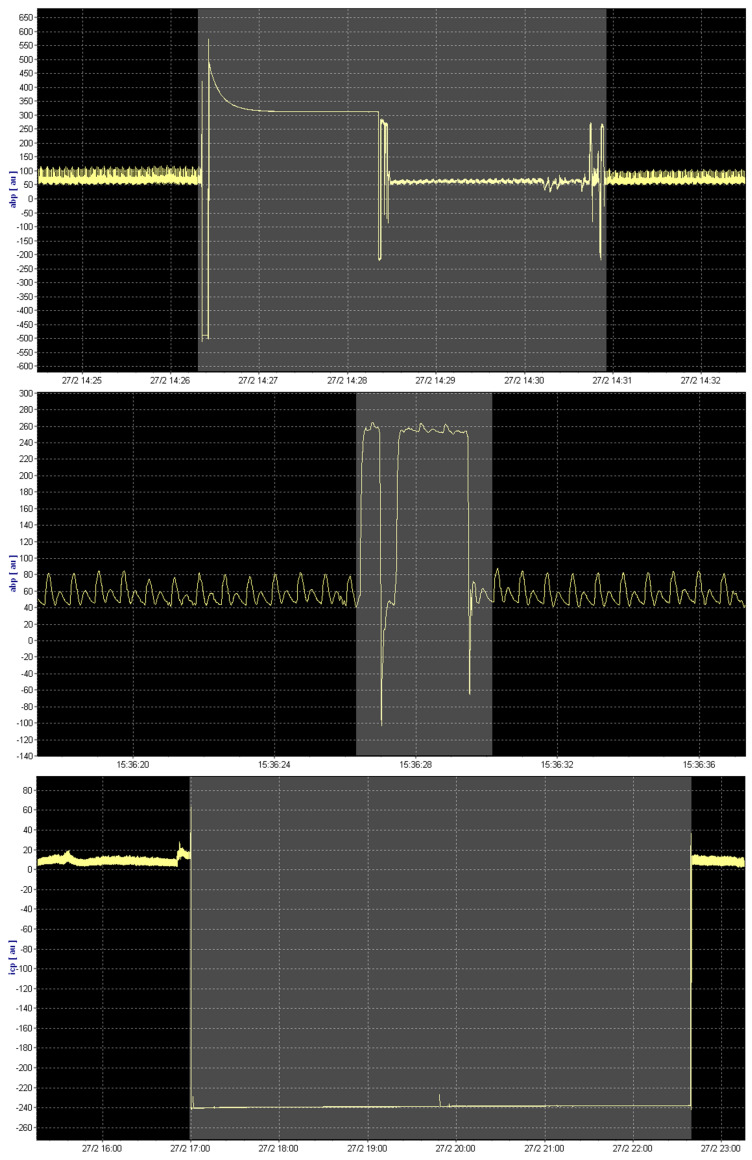
Data artifact removal examples. Grayed-out regions represent data segments that were identified as artifacts and removed accordingly. Figure was produced using ICM+ software. abp = arterial blood pressure, icp = intracranial pressure.

**Figure A2:**
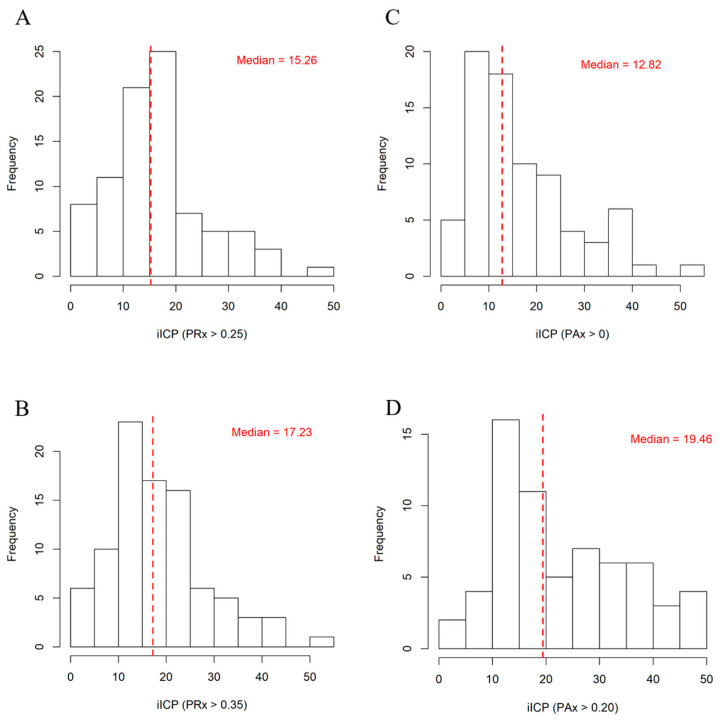
Patient cohort cerebral physiology. (**A**) iICP derived using PRx > 0.25. (**B**) iICP derived using PRx > 0.35. (**C**) iICP derived using PAx > 0. (**D**) iICP derived using PAx > 0.20. Red dashed lines demarcate median values. iICP = individualized intracranial pressure threshold, PAx = pulse amplitude index, PRx = pressure reactivity index, RAC = correlation between slow waves of AMP and CPP.

**Figure A3:**
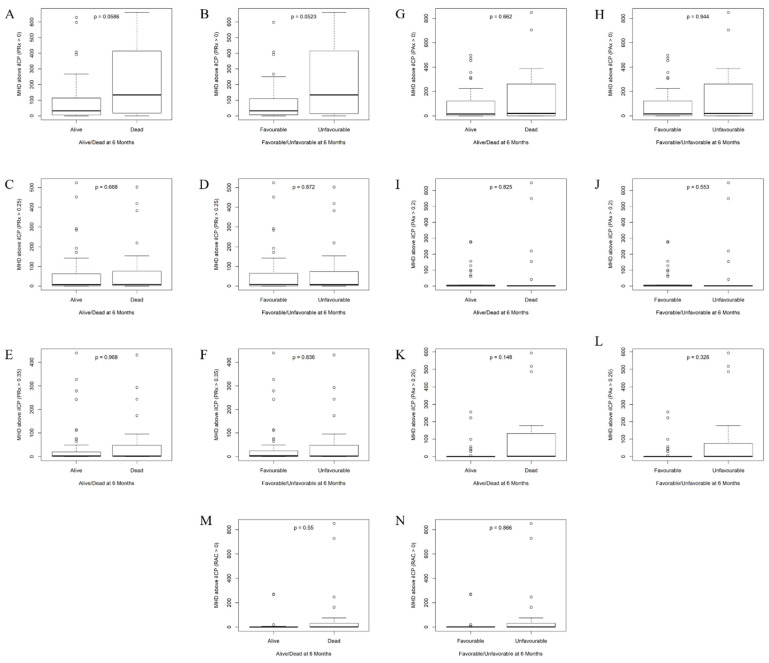
Box plots of mean hourly doses of ICP above iICP thresholds for Alive/Dead and Favorable/Unfavorable at 6 months. (**A**) Mean hourly dose of ICP above iICP (PRx > 0) for Alive/Dead. (**B**) Mean hourly dose of ICP above iICP (PRx > 0) for Favorable/Unfavorable. (**C**) Mean hourly dose of ICP above iICP (PRx > 0.25) for Alive/Dead. (**D**) Mean hourly dose of ICP above iICP (PRx > 0.25) for Favorable/Unfavorable. (**E**) Mean hourly dose of ICP above iICP (PRx > 0.35) for Alive/Dead. (**F**) Mean hourly dose of ICP above iICP (PRx > 0.35) for Favorable/Unfavorable. (**G**) Mean hourly dose of ICP above iICP (PAx > 0) for Alive/Dead. (**H**) Mean hourly dose of ICP above iICP (PAx > 0) for Favorable/Unfavorable. (**I**) Mean hourly dose of ICP above iICP (PAx > 0.20) for Alive/Dead. (**J**) Mean hourly dose of ICP above iICP (PAx > 0.20) for Favorable/Unfavorable. (**K**) Mean hourly dose of ICP above iICP (PAx > 0.25) for Alive/Dead. (**L**) Mean hourly dose of ICP above iICP (PAx > 0.25) for Favorable/Unfavorable. (**M**) Mean hourly dose of ICP above iICP (RAC > 0) for Alive/Dead. (**N**) Mean hourly dose of ICP above iICP (RAC > 0) for Favorable/Unfavorable. Alive (GOSE 2–8), Dead (GOSE 1), Favorable (GOSE 5–8), Unfavorable (GOSE 1–4). *p*-values were calculated using the Mann–Whitney U test. iICP = individualized intracranial pressure threshold, MHD = mean hourly dose, PAx = pulse amplitude index, PRx = pressure reactivity index, RAC = correlation (R) between slow waves of AMP (A) and CPP (C).

**Figure A4:**
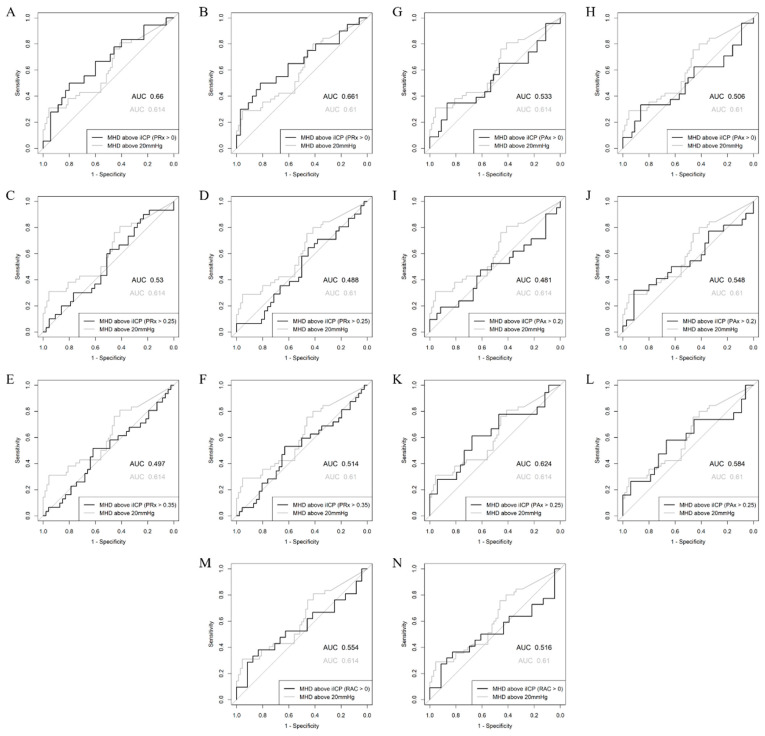
Univariate logistic regression ROC curves for mean hourly doses of ICP above iICP thresholds. (**A**) Mean hourly dose of ICP above iICP (PRx > 0) for Alive/Dead. (**B**) Mean hourly dose of ICP above iICP (PRx > 0) for Favorable/Unfavorable. (**C**) Mean hourly dose of ICP above iICP (PRx > 0.25) for Alive/Dead. (**D**) Mean hourly dose of ICP above iICP (PRx > 0.25) for Favorable/Unfavorable. (**E**) Mean hourly dose of ICP above iICP (PRx > 0.35) for Alive/Dead. (**F**) Mean hourly dose of ICP above iICP (PRx > 0.35) for Favorable/Unfavorable. (**G**) Mean hourly dose of ICP above iICP (PAx > 0) for Alive/Dead. (**H**) Mean hourly dose of ICP above iICP (PAx > 0) for Favorable/Unfavorable. (**I**) Mean hourly dose of ICP above iICP (PAx > 0.20) for Alive/Dead. (**J**) Mean hourly dose of ICP above iICP (PAx > 0.20) for Favorable/Unfavorable. (**K**) Mean hourly dose of ICP above iICP (PAx > 0.25) for Alive/Dead. (**L**) Mean hourly dose of ICP above iICP (PAx > 0.25) for Favorable/Unfavorable. (**M**) Mean hourly dose of ICP above iICP (RAC > 0) for Alive/Dead. (**N**) Mean hourly dose of ICP above iICP (RAC > 0) for Favorable/Unfavorable. Alive (GOSE 2–8), Dead (GOSE 1), Favorable (GOSE 5–8), Unfavorable (GOSE 1–4). AUC = area under the curve, iICP = individualized intracranial pressure threshold, MHD = mean hourly dose, PAx = pulse amplitude index, PRx = pressure reactivity index, RAC = correlation (R) between slow waves of AMP (A) and CPP (C).

## Data Availability

The datasets analyzed in this study are not publicly available due to patient privacy restrictions but can be requested from the senior author at frederick.zeiler@umanitoba.ca.

## Abbreviations

The following abbreviations are used in this manuscript:

ABP	arterial blood pressure
AIC	Akaike information criterion
AMP	pulse amplitude of ICP
a.u.	arbitrary units
AUC	area under the curve
BTF	Brain Trauma Foundation
CI	confidence interval
CPP	cerebral perfusion pressure
CPPopt	cerebral perfusion pressure optimum
CT	computed tomography
EVD	external ventricular drain
GCS	Glasgow Coma Scale
GOSE	Glasgow Outcome Scale-Extended
ICM+	Intensive Care Monitoring “Plus”
ICP	intracranial pressure
SICU	surgical intensive care unit
iICP	individualized intracranial pressure threshold
iICP.ci	iICP values filtered for those associated with a 95% CI of less than 0.2 a.u.
iICP.5-25	iICP values filtered for those within a range of 5–25 mmHg
IMPACT	International Mission for Prognosis and Analysis of Clinical Trials
IQR	interquartile range
LOESS	locally weighted scatterplot smoothing
MAP	mean arterial pressure
mmHg	millimeters of mercury
PAx	pulse amplitude index (correlation between AMP and MAP)
PRx	pressure reactivity index (correlation between ICP and MAP)
RAC	correlation (R) between AMP (A) and CPP (C)

## Appendix B

**Table A1:** Patient cohort demographics.

Demographic Variable			Median (IQR) or Raw Numbers (%)
Number of Patients			124
Age (years)			42 (27–57)
Sex	Male		103 (83%)
	Female		21 (17%)
Admission GCS Total			6.5 (4–8)
Admission GCS Motor			4 (2–5)
Admission GCS Eyes			1 (1–2)
Admission GCS Verbal			1 (1–1)
Admission Pupillary Response		Bilaterally Reactive	73 (59%)
		Unilaterally Unreactive	32 (26%)
		Bilaterally Unreactive	19 (15%)
Marshall CT Grade			5 (3–5)
Rotterdam CT Grade			4 (4–5.25)
Helsinki CT Score			6 (4.75–9)
Stockholm CT Score			3.2 (2.5–3.9)
GOSE (6 Months)			5 (1–7)
Number Alive (GOSE 2–8) at 6 Months			68 (62%)
Number Dead (GOSE 1) at 6 Months			42 (38%)
Number Favorable (GOSE 5–8) at 6 Months			65 (59%)
Number Unfavorable (GOSE 1–4) at 6 Months			45 (41%)
Number with Hypoxia Episode			40 (32%)
Number with Hypotension Episode			12 (10%)
Number with Traumatic SAH			120 (97%)
Number with Epidural Hematoma			13 (10%)
Admission Hemoglobin			133.5 (115–146.75)
Admission Serum Glucose			8.2 (7.1–11.1)
Length of Hospital Stay			21 (8.5–42)
Length of ICU Stay			8 (4–15)

CT = computed tomography, GCS = Glasgow Coma Scale, GOSE = Glasgow Outcome Scale-Extended, ICU = intensive care unit, IQR = interquartile range, SAH = subarachnoid hemorrhage.

## Appendix C

**Table A2:** Patient cohort cerebral physiology.

Physiologic Metric	Median (IQR)
Duration of Physiologic Monitoring (hours)	73.27 (37.17–130.3)
Mean MAP (mmHg)	84.35 (79.72–88.66)
Mean ICP (mmHg)	9.418 (5.806–12.7)
% Time ICP > 20 mmHg	1.476 (0.07516–5.751)
Mean Hourly Dose of ICP > 20 mmHg	2.958 (0.05611–12.25)
% Time ICP > 22 mmHg	0.7846 (0–2.968)
Mean Hourly Dose of ICP > 22 mmHg	1.636 (0–8.123)
Mean CPP (mmHg)	73.93 (70.2–79.62)
% Time CPP < 60 mmHg	4.031 (1.114–8.496)
% Time CPP > 70 mmHg	64.06 (46.42–76.28)
Mean PRx	0.1545 (0.03913–0.2809)
% Time PRx > 0	66.27 (52.7–81.71)
% Time PRx > 0.25	41.36 (27.82–57.17)
% Time PRx > 0.35	31.36 (19.48–46.01)
Mean PAx	0.004365 (−0.1267–0.1265)
% Time PAx > 0	50.9 (34.85–66.36)
% Time PAx > 0.20	30.08 (15.91–45.56)
% Time PAx > 0.25	25.26 (12.43–40.13)
Mean RAC	−0.2301 (−0.3928–−0.03803)
% Time RAC > 0	24.47 (14–44.41)
iICP (PRx > 0)	10.98 (5.94–18.31)
% Time ICP > iICP (PRx > 0)	19.05 (3.106–74.89)
Mean Hourly Dose of ICP > iICP (PRx > 0)	50.31 (6.53–230.5)
iICP (PRx > 0.25)	15.26 (10.57–19.95)
% Time ICP > iICP (PRx > 0.25)	3.411 (0.4349–23.99)
Mean Hourly Dose of ICP > iICP (PRx > 0.25)	7.491 (1.065–63.18)
iICP (PRx > 0.35)	17.23 (11.59–22.77)
% Time ICP > iICP (PRx > 0.35)	1.175 (0.329–9.568)
Mean Hourly Dose of ICP > iICP (PRx > 0.35)	2.764 (0.6038–24.97)
iICP (PAx > 0)	12.82 (8.985–21.27)
% Time ICP > iICP (PAx > 0)	3.537 (0.5514–65.18)
Mean Hourly Dose of ICP > iICP (PAx > 0)	14.58 (0.8713–147.2)
iICP (PAx > 0.20)	19.46 (13.58–32.49)
% Time ICP > iICP (PAx > 0.20)	0.4343 (0.1083–4.008)
Mean Hourly Dose of ICP > iICP (PAx > 0.20)	1.023 (0.2026–6.59)
iICP (PAx > 0.25)	19.91 (14.79–35.46)
% Time ICP > iICP (PAx > 0.25)	0.334 (0.1015–1.53)
Mean Hourly Dose of ICP > iICP (PAx > 0.25)	0.74 (0.2006–4.963)
iICP (RAC > 0)	18.7 (11.9–25.76)
% Time ICP > iICP (RAC > 0)	0.6271 (0.1819–3.758)
Mean Hourly Dose of ICP > iICP (RAC > 0)	1.067 (0.132–6.579)
Duration of Physiologic Monitoring (hours)	73.27 (37.17–130.3)
Mean MAP (mmHg)	84.35 (79.72–88.66)
Mean ICP (mmHg)	9.418 (5.806–12.7)
% Time ICP > 20 mmHg	1.476 (0.07516–5.751)
Mean Hourly Dose of ICP > 20 mmHg	2.958 (0.05611–12.25)
% Time ICP > 22 mmHg	0.7846 (0–2.968)
Mean Hourly Dose of ICP > 22 mmHg	1.636 (0–8.123)
Mean CPP (mmHg)	73.93 (70.2–79.62)
% Time CPP < 60 mmHg	4.031 (1.114–8.496)
% Time CPP > 70 mmHg	64.06 (46.42–76.28)
Mean PRx	0.1545 (0.03913–0.2809)
% Time PRx > 0	66.27 (52.7–81.71)
% Time PRx > 0.25	41.36 (27.82–57.17)
% Time PRx > 0.35	31.36 (19.48–46.01)
Mean PAx	0.004365 (−0.1267–0.1265)
% Time PAx > 0	50.9 (34.85–66.36)
% Time PAx > 0.20	30.08 (15.91–45.56)
% Time PAx > 0.25	25.26 (12.43–40.13)
Mean RAC	−0.2301 (−0.3928–−0.03803)
% Time RAC > 0	24.47 (14–44.41)
iICP (PRx > 0)	10.98 (5.94–18.31)
% Time ICP > iICP (PRx > 0)	19.05 (3.106–74.89)
Mean Hourly Dose of ICP > iICP (PRx > 0)	50.31 (6.53–230.5)
iICP (PRx > 0.25)	15.26 (10.57–19.95)
% Time ICP > iICP (PRx > 0.25)	3.411 (0.4349–23.99)
Mean Hourly Dose of ICP > iICP (PRx > 0.25)	7.491 (1.065–63.18)
iICP (PRx > 0.35)	17.23 (11.59–22.77)
% Time ICP > iICP (PRx > 0.35)	1.175 (0.329–9.568)
Mean Hourly Dose of ICP > iICP (PRx > 0.35)	2.764 (0.6038–24.97)
iICP (PAx > 0)	12.82 (8.985–21.27)
% Time ICP > iICP (PAx > 0)	3.537 (0.5514–65.18)
Mean Hourly Dose of ICP > iICP (PAx > 0)	14.58 (0.8713–147.2)
iICP (PAx > 0.20)	19.46 (13.58–32.49)
% Time ICP > iICP (PAx > 0.20)	0.4343 (0.1083–4.008)
Mean Hourly Dose of ICP > iICP (PAx > 0.20)	1.023 (0.2026–6.59)
iICP (PAx > 0.25)	19.91 (14.79–35.46)
% Time ICP > iICP (PAx > 0.25)	0.334 (0.1015–1.53)
Mean Hourly Dose of ICP > iICP (PAx > 0.25)	0.74 (0.2006–4.963)
iICP (RAC > 0)	18.7 (11.9–25.76)
% Time ICP > iICP (RAC > 0)	0.6271 (0.1819–3.758)
Mean Hourly Dose of ICP > iICP (RAC > 0)	1.067 (0.132–6.579)

CPP = cerebral perfusion pressure, ICP = intracranial pressure, iICP = individualized intracranial pressure threshold, IQR = interquartile range, MAP = mean arterial pressure, mmHg = millimeters of mercury, PAx = pulse amplitude index, PRx = pressure reactivity index, RAC = correlation (R) between slow waves of AMP (A) and CPP (C).

## Appendix F

**Table A3:** Mann–Whitney U/Chi-square testing for iICP (PRx > 0) identified vs. not identified.

Variable	Identified Median (IQR)	Not Identified Median (IQR)	*p*-Value
Age (years)	36 (25–59)	44 (34–54.5)	0.136
Sex (% Male)	80.30%	85.70%	0.575
Admission GCS Total	7 (4–8)	6 (4–8)	0.653
Admission GCS Motor	4 (2–5)	4 (2–5)	0.197
Admission GCS Eyes	1 (1–2)	1 (1–2)	0.664
Admission GCS Verbal	1 (1–1)	1 (1–2)	0.454
Admission Pupil Response (% Bilaterally Reactive)	60.70%	57.10%	0.924
Marshall CT Grade	4 (3–5)	5 (3–5)	0.685
Rotterdam CT Grade	4 (4–5)	5 (4–5.5)	0.756
Helsinki CT Score	6 (4–9)	6 (5–9)	0.880
Stockholm CT Score	3.3 (2.5–4)	3.2 (2.45–3.75)	0.532
GOSE (6 Months)	5 (1–7)	5 (1–7)	0.841
Number Alive (GOSE 2–8) at 6 Months	66%	57.90%	0.495
Number Dead (GOSE 1) at 6 Months	34%	42.10%	0.495
Number Favorable (GOSE 5–8) at 6 Months	62.30%	56.10%	0.646
Number Unfavorable (GOSE 1–4) at 6 Months	37.70%	43.90%	0.646
Number with Hypoxia Episode	29.50%	34.90%	0.651
Number with Hypotension Episode	4.90%	14.30%	0.144
Number with Traumatic SAH	96.70%	96.80%	1.000
Number with Epidural Hematoma	9.80%	11.10%	1.000
Admission Hemoglobin	135 (116–148)	133 (114–146)	0.720
Admission Serum Glucose	8.15 (7.38–11.9)	8.4 (6.9–11)	0.465
Length of Hospital Stay	27 (10–46)	20.5 (8–34.8)	0.177
Length of ICU Stay	10.5 (5–18.8)	7 (4–11)	**0.014**
Mean MAP (mmHg)	82.6 (80–86.5)	86 (79–90.5)	0.210
Mean ICP (mmHg)	10.5 (6.11–13)	8.55 (5.13–11.9)	0.372
Mean Hourly Dose of ICP > 20 mmHg	4.18 (0.239–11.8)	1.21 (0.015–14)	0.320
Mean Hourly Dose of ICP > 22 mmHg	2.32 (0.0213–8)	0.671 (0–7.68)	0.375
Mean CPP (mmHg)	73.6 (71.1–76.6)	75.1 (69.5–80.5)	0.359
% Time CPP < 60 mmHg	5.08 (1.71–9.26)	2.82 (0.48–7.31)	0.108
% Time CPP > 70 mmHg	59.1 (47.5–73.4)	68.4 (40.6–82.6)	0.433
Mean PRx	0.0396 (−0.0607–0.138)	0.272 (0.179–0.405)	**<0.001**
% Time PRx > 0	53.2 (41.7–64.5)	81.5 (68.9–88.4)	**<0.001**
% Time PRx > 0.25	28.3 (20.9–39.4)	57.1 (42.6–71.4)	**<0.001**
% Time PRx > 0.35	19.5 (13.1–29.2)	44.2 (31.9–61.4)	**<0.001**
Mean PAx	−0.09 (−0.172–0.0342)	0.0638 (−0.0418–0.241)	**<0.001**
% Time PAx > 0	38.6 (29.7–55.6)	59.2 (43.9–76.4)	**<0.001**
% Time PAx > 0.20	21.2 (12.1–33.7)	37 (23–58.3)	**<0.001**
% Time PAx > 0.25	17.7 (9.68–28.5)	30.6 (18.3–51.9)	**<0.001**
Mean RAC	−0.376 (−0.47–−0.195)	−0.123 (−0.27–0.0155)	**<0.001**
% Time RAC > 0	16.3 (8.21–29.1)	35.7 (22.1–52.3)	**<0.001**

Bolded *p*-values are those reaching statistical significance, *p* < 0.05. CPP = cerebral perfusion pressure, CT = computed tomography, GCS = Glasgow Coma Scale, ICP = intracranial pressure, ICU = intensive care unit, IQR = interquartile range, MAP = mean arterial pressure, mmHg = millimeters of mercury, PAx = pulse amplitude index, PRx = pressure reactivity index, SAH = subarachnoid hemorrhage, RAC = correlation (R) between slow waves of AMP (A) and CPP (C).

## Appendix G

**Table A4:** Mann–Whitney U/Chi-square testing for iICP (PRx > 0.25) identified vs. not identified.

Variable	Identified Median (IQR)	Not Identified Median (IQR)	*p*-Value
Age (years)	42 (27–57)	40.5 (28.2–52.8)	0.651
Sex (% Male)	83.70%	81.60%	0.973
Admission GCS Total	6 (4–8)	7 (4.25–9.75)	0.213
Admission GCS Motor	4 (2–5)	4 (2–5)	0.471
Admission GCS Eyes	1 (1–2)	1 (1–2)	0.172
Admission GCS Verbal	1 (1–1)	1 (1–2)	**0.004**
Admission Pupil Response (% Bilaterally Reactive)	54.70%	68.40%	0.233
Marshall CT Grade	5 (3–5)	4 (3–5)	0.673
Rotterdam CT Grade	4.5 (4–6)	4 (4–5)	0.804
Helsinki CT Score	7 (5–9)	5 (4–9)	0.375
Stockholm CT Score	3.25 (2.58–3.98)	3 (2.4–3.85)	0.397
GOSE (6 Months)	5 (1–7)	7 (1–7)	0.177
Number Alive (GOSE 2–8) at 6 Months	58.90%	67.60%	0.499
Number Dead (GOSE 1) at 6 Months	41.10%	32.40%	0.499
Number Favorable (GOSE 5–8) at 6 Months	57.50%	62.20%	0.794
Number Unfavorable (GOSE 1–4) at 6 Months	42.50%	37.80%	0.794
Number with Hypoxia Episode	29.10%	39.50%	0.350
Number with Hypotension Episode	5.80%	18.40%	0.063
Number with Traumatic SAH	96.50%	97.40%	1.000
Number with Epidural Hematoma	10.50%	10.50%	1.000
Admission Hemoglobin	134 (116–147)	133 (113–145)	0.599
Admission Serum Glucose	8.3 (7.05–11.6)	8.05 (7.12–9.8)	0.384
Length of Hospital Stay	21 (10–42)	21 (7.25–35.8)	0.309
Length of ICU Stay	9 (4–15.2)	7 (3.25–12.5)	0.133
Mean MAP (mmHg)	84 (79.9–88.4)	85.7 (79.7–89.6)	0.680
Mean ICP (mmHg)	10.1 (6.27–12.4)	8.03 (4.52–14.8)	0.823
Mean Hourly Dose of ICP > 20 mmHg	2.78 (0.0431–10.8)	3.11 (0.0817–43.3)	0.567
Mean Hourly Dose of ICP > 22 mmHg	1.64 (0–7.55)	1.56 (0.00381–32.7)	0.636
Mean CPP (mmHg)	73.9 (71.1–79.3)	74.1 (68.9–79.9)	0.798
% Time CPP < 60 mmHg	3.96 (1.18–8.57)	4.61 (0.523–8.41)	0.933
% Time CPP > 70 mmHg	64.1 (46–76.7)	64 (48–75.4)	0.835
Mean PRx	0.149 (0.0381–0.224)	0.228 (0.0491–0.416)	**0.026**
% Time PRx > 0	64.6 (52.5–71.7)	78.2 (55.9–88.3)	**0.008**
% Time PRx > 0.25	39.7 (27.8–50.8)	51.5 (28.3–73.6)	**0.020**
% Time PRx > 0.35	29.8 (19.1–41)	37.1 (20–63.8)	**0.028**
Mean PAx	−0.0205 (−0.146–0.123)	0.023 (−0.0966–0.149)	0.160
% Time PAx > 0	46.6 (33–65.1)	53.1 (38.3–66.8)	0.172
% Time PAx > 0.20	29.7 (15.3–45.3)	31.3 (21.9–48.3)	0.213
% Time PAx > 0.25	24.4 (11.8–39.7)	26.8 (17.2–42.5)	0.203
Mean RAC	−0.26 (−0.417–−0.0831)	−0.199 (−0.319–0.00956)	**0.047**
% Time RAC > 0	22.7 (11.9–39.4)	27.7 (19.1–50.4)	0.060

Bolded *p*-values are those reaching statistical significance, *p* < 0.05. CPP = cerebral perfusion pressure, CT = computed tomography, GCS = Glasgow Coma Scale, ICP = intracranial pressure, ICU = intensive care unit, IQR = interquartile range, MAP = mean arterial pressure, mmHg = millimeters of mercury, PAx = pulse amplitude index, PRx = pressure reactivity index, SAH = subarachnoid hemorrhage, RAC = correlation (R) between slow waves of AMP (A) and CPP (C).

## Appendix H

**Table A5:** Mann–Whitney U/Chi-square testing for iICP (PRx > 0.35) identified vs. not identified.

Variable	Identified Median (IQR)	Not Identified Median (IQR)	*p*-Value
Age (years)	42 (26.2–57)	41 (29.8–52.8)	0.935
Sex (% Male)	86.70%	73.50%	0.141
Admission GCS Total	6 (4–8)	7 (5–8.75)	0.200
Admission GCS Motor	4 (2–5)	4.5 (2.25–5)	0.256
Admission GCS Eyes	1 (1–2)	1 (1–2)	0.343
Admission GCS Verbal	1 (1–1)	1 (1–2)	**0.048**
Admission Pupil Response (% Bilaterally Reactive)	55.60%	67.60%	**0.014**
Marshall CT Grade	5 (3–5)	4.5 (3.25–5)	0.816
Rotterdam CT Grade	4.5 (4–5.75)	4 (4–5)	0.961
Helsinki CT Score	6 (5–9)	5.5 (4.25–9)	0.998
Stockholm CT Score	3.25 (2.5–3.9)	3 (2.4–3.85)	0.494
GOSE (6 Months)	5 (1–7)	6 (1–7)	0.623
Number Alive (GOSE 2–8) at 6 Months	60.30%	65.60%	0.756
Number Dead (GOSE 1) at 6 Months	39.70%	34.40%	0.756
Number Favorable (GOSE 5–8) at 6 Months	59%	59.40%	1.000
Number Unfavorable (GOSE 1–4) at 6 Months	41%	40.60%	1.000
Number with Hypoxia Episode	28.90%	41.20%	0.276
Number with Hypotension Episode	8.90%	11.80%	0.886
Number with Traumatic SAH	96.70%	97.10%	1.000
Number with Epidural Hematoma	8.90%	14.70%	0.539
Admission Hemoglobin	134 (117–148)	130 (111–142)	0.161
Admission Serum Glucose	8.4 (7–11.4)	8.05 (7.12–10)	0.515
Length of Hospital Stay	21 (10–50.2)	22 (7–32)	0.137
Length of ICU Stay	9.5 (4–15.2)	7 (3–9.75)	**0.030**
Mean MAP (mmHg)	84 (79.2–88.4)	85.6 (81–89.6)	0.571
Mean ICP (mmHg)	9.79 (5.35–12.5)	8.51 (6.24–14.5)	0.460
Mean Hourly Dose of ICP > 20 mmHg	2.65 (0.0102–10.8)	3.3 (0.35–32.7)	0.271
Mean Hourly Dose of ICP > 22 mmHg	1.59 (0–7.55)	1.94 (0.192–19.8)	0.329
Mean CPP (mmHg)	74.2 (71.1–79.7)	73.6 (68.5–78.4)	0.282
% Time CPP < 60 mmHg	4.03 (1.13–7.94)	4.24 (0.577–9.57)	0.799
% Time CPP > 70 mmHg	64.7 (48.2–77.1)	63 (36.8–73.4)	0.381
Mean PRx	0.153 (0.0446–0.269)	0.164 (0.00939–0.416)	0.463
% Time PRx > 0	65.3 (53.4–79.5)	71.3 (51.8–88.3)	0.216
% Time PRx > 0.25	40.5 (28.7–56.4)	42.2 (24.3–74.1)	0.392
% Time PRx > 0.35	31.8 (19.5–42.4)	29.8 (18.1–63.8)	0.534
Mean PAx	−0.0205 (−0.146–0.123)	0.0249 (−0.0729–0.269)	0.099
% Time PAx > 0	46.6 (33–65.3)	53.2 (39.6–78.2)	0.113
% Time PAx > 0.20	30.1 (15.3–45.3)	30.2 (21.9–61.4)	0.204
% Time PAx > 0.25	25.3 (11.8–39.7)	25.2 (17.2–55.9)	0.200
Mean RAC	−0.241 (−0.38–−0.0612)	−0.209 (−0.402–0.0125)	0.344
% Time RAC > 0	24.1 (13.7–42.2)	26.3 (14.9–54.3)	0.386

Bolded *p*-values are those reaching statistical significance, *p* < 0.05. CPP = cerebral perfusion pressure, CT = computed tomography, GCS = Glasgow Coma Scale, ICP = intracranial pressure, ICU = intensive care unit, IQR = interquartile range, MAP = mean arterial pressure, mmHg = millimeters of mercury, PAx = pulse amplitude index, PRx = pressure reactivity index, SAH = subarachnoid hemorrhage, RAC = correlation (R) between slow waves of AMP (A) and CPP (C).

## Appendix I

**Table A6:** Mann–Whitney U/chi-square testing for iICP (PAx > 0) identified vs. not identified.

Variable	Identified Median (IQR)	Not Identified Median (IQR)	*p*-Value
Age (years)	38 (25–50)	51 (32.5–60)	**0.020**
Sex (% Male)	83.10%	83%	1.000
Admission GCS Total	7 (4–8)	6 (4–8)	0.941
Admission GCS Motor	4 (2–5)	4 (2–5)	0.515
Admission GCS Eyes	1 (1–2)	1 (1–2)	0.459
Admission GCS Verbal	1 (1–1)	1 (1–2)	0.061
Admission Pupil Response (% Bilaterally Reactive)	62.30%	53.20%	0.098
Marshall CT Grade	4 (3–5)	5 (3.5–5)	0.443
Rotterdam CT Grade	4 (3–6)	5 (4–5)	0.522
Helsinki CT Score	6 (4–9)	6 (5–9)	0.646
Stockholm CT Score	3.2 (2.5–3.95)	3.2 (2.5–3.75)	0.928
GOSE (6 Months)	6 (1–7)	4 (1–7)	0.367
Number Alive (GOSE 2–8) at 6 Months	66.20%	54.80%	0.320
Number Dead (GOSE 1) at 6 Months	33.80%	45.20%	0.320
Number Favorable (GOSE 5–8) at 6 Months	64.70%	50%	0.185
Number Unfavorable (GOSE 1–4) at 6 Months	35.30%	50%	0.185
Number with Hypoxia Episode	29.90%	36.20%	0.596
Number with Hypotension Episode	6.50%	14.90%	0.222
Number with Traumatic SAH	96.10%	97.90%	0.987
Number with Epidural Hematoma	5.20%	19.10%	**0.031**
Admission Hemoglobin	134 (118–148)	130 (113–145)	0.417
Admission Serum Glucose	8.4 (7.05–11.6)	7.95 (7.1–10.4)	0.625
Length of Hospital Stay	22 (10–42)	18.5 (7.25–38)	0.283
Length of ICU Stay	8.5 (4–17)	7.5 (4–13.8)	0.305
Mean MAP (mmHg)	84.8 (80.4–89.4)	82.7 (79–87)	0.170
Mean ICP (mmHg)	10.9 (6.84–13.3)	7.9 (3.9–10.6)	**0.016**
Mean Hourly Dose of ICP > 20 mmHg	5.75 (0.0626–16.9)	1.05 (0.0343–7.77)	0.089
Mean Hourly Dose of ICP > 22 mmHg	3.1 (0.0105–9.22)	0.389 (0–3.62)	0.080
Mean CPP (mmHg)	74.6 (71.2–79.7)	73.6 (69.4–78.4)	0.369
% Time CPP < 60 mmHg	4.51 (1.13–9.26)	3.19 (0.902–7.73)	0.392
% Time CPP > 70 mmHg	64.2 (50.4–75.9)	63.9 (39–76)	0.564
Mean PRx	0.148 (0.0109–0.265)	0.192 (0.12–0.383)	**0.016**
% Time PRx > 0	63 (51.7–79.2)	70.1 (63.1–86.7)	**0.008**
% Time PRx > 0.25	39.4 (26.8–56.7)	45.8 (34.4–70.5)	**0.018**
% Time PRx > 0.35	29.2 (18.3–42.3)	35.2 (25.3–59.3)	**0.028**
Mean PAx	−0.0369 (−0.127–0.0638)	0.116 (−0.107–0.28)	**0.003**
% Time PAx > 0	45.1 (33.7–59.2)	64.9 (37.5–82.4)	**0.002**
% Time PAx > 0.20	24.2 (15.3–36.7)	41.4 (19.4–63.8)	**0.003**
% Time PAx > 0.25	20.7 (12.4–30.8)	35.1 (16.3–57.8)	**0.003**
Mean RAC	−0.288 (−0.437–−0.125)	−0.122 (−0.345–0.0413)	**<0.001**
% Time RAC > 0	21.4 (11.4–34)	36.1 (19.5–56)	**<0.001**

Bolded *p*-values are those reaching statistical significance, *p* < 0.05. CPP = cerebral perfusion pressure, CT = computed tomography, GCS = Glasgow Coma Scale, ICP = intracranial pressure, ICU = intensive care unit, IQR = interquartile range, MAP = mean arterial pressure, mmHg = millimeters of mercury, PAx = pulse amplitude index, PRx = pressure reactivity index, SAH = subarachnoid hemorrhage, RAC = correlation (R) between slow waves of AMP (A) and CPP (C).

## Appendix J

**Table A7:** Mann–Whitney U/chi-square testing for iICP (PAx > 0.20) identified vs. not identified.

Variable	Identified Median (IQR)	Not Identified Median (IQR)	*p*-Value
Age (years)	42 (31–56.2)	40.5 (24–57)	0.562
Sex (% Male)	84.40%	81.70%	0.871
Admission GCS Total	6.5 (4–8)	6.5 (4–8)	0.946
Admission GCS Motor	4 (2–5)	4 (2–5)	0.733
Admission GCS Eyes	1 (1–2)	1 (1–2)	0.498
Admission GCS Verbal	1 (1–1)	1 (1–1.25)	0.826
Admission Pupil Response (% Bilaterally Reactive)	56.20%	61.70%	0.548
Marshall CT Grade	5 (3–5)	4.5 (3–5)	0.783
Rotterdam CT Grade	4.5 (3–5.25)	4 (4–5.25)	0.762
Helsinki CT Score	6.5 (4–9)	6 (5–9)	0.860
Stockholm CT Score	3.25 (2.88–3.83)	3.2 (2.4–3.92)	0.702
GOSE (6 Months)	5 (1–7)	5 (1–7)	0.771
Number Alive (GOSE 2–8) at 6 Months	63.20%	60.40%	0.918
Number Dead (GOSE 1) at 6 Months	36.80%	39.60%	0.918
Number Favorable (GOSE 5–8) at 6 Months	61.40%	56.60%	0.751
Number Unfavorable (GOSE 1–4) at 6 Months	38.60%	43.40%	0.751
Number with Hypoxia Episode	26.60%	38.30%	0.227
Number with Hypotension Episode	6.20%	13.30%	0.303
Number with Traumatic SAH	98.40%	95%	0.566
Number with Epidural Hematoma	9.40%	11.70%	0.902
Admission Hemoglobin	133 (122–146)	134 (113–148)	0.623
Admission Serum Glucose	8.4 (7.1–10.8)	7.95 (7.07–11.1)	0.719
Length of Hospital Stay	26 (11–42)	15 (7–40)	0.085
Length of ICU Stay	11 (5.5–17)	7 (4–13)	0.022
Mean MAP (mmHg)	84.6 (80.8–89.2)	82.8 (79–87.5)	0.206
Mean ICP (mmHg)	10.2 (6.95–13.1)	8.39 (4.85–12.2)	0.387
Mean Hourly Dose of ICP > 20 mmHg	5.21 (0.0561–12.3)	2.43 (0.0514–12.9)	0.517
Mean Hourly Dose of ICP > 22 mmHg	2.83 (0.00789–8.12)	0.933 (0–6.9)	0.424
Mean CPP (mmHg)	75 (71.2–79.6)	73.3 (69.6–79.1)	0.163
% Time CPP < 60 mmHg	4.03 (1.03–7.73)	4.17 (1.43–10.1)	0.812
% Time CPP > 70 mmHg	67.7 (52.1–78.2)	62.1 (42.4–74.1)	0.277
Mean PRx	0.153 (0.0316–0.272)	0.158 (0.0633–0.291)	0.620
% Time PRx > 0	66 (52.3–80.1)	67.4 (55.7–83.8)	0.515
% Time PRx > 0.25	40.1 (27.3–56.8)	41.7 (28.8–58.9)	0.532
% Time PRx > 0.35	31.1 (18.9–44.5)	31.6 (20.1–46.4)	0.578
Mean PAx	0.0293 (−0.109–0.117)	−0.0202 (−0.128–0.162)	0.942
% Time PAx > 0	53.4 (35.3–63.1)	47.8 (34.7–67.6)	0.848
% Time PAx > 0.20	32.4 (15.9–44.9)	28.1 (15.9–47.1)	0.998
% Time PAx > 0.25	27 (12.4–39)	23.5 (12.4–41.9)	0.986
Mean RAC	−0.23 (−0.409–−0.0751)	−0.23 (−0.379–−0.0237)	0.496
% Time RAC > 0	24.5 (12.5–40.4)	23.8 (15.6–45.7)	0.532

Bolded *p*-values are those reaching statistical significance, *p* < 0.05. CPP = cerebral perfusion pressure, CT = computed tomography, GCS = Glasgow Coma Scale, ICP = intracranial pressure, ICU = intensive care unit, IQR = interquartile range, MAP = mean arterial pressure, mmHg = millimeters of mercury, PAx = pulse amplitude index, PRx = pressure reactivity index, SAH = subarachnoid hemorrhage, RAC = correlation (R) between slow waves of AMP (A) and CPP (C).

## Appendix K

**Table A8:** Mann–Whitney U/chi-square testing for iICP (PAx > 0.25) identified vs. not identified.

Variable	Identified Median (IQR)	Not Identified Median (IQR)	*p*-Value
Age (years)	42 (28–54.5)	41.5 (25.5–57.2)	0.990
Sex (% Male)	81.70%	84.40%	0.871
Admission GCS Total	6.5 (4–8)	6.5 (4–8)	0.758
Admission GCS Motor	4 (2–5)	4 (2–5)	0.657
Admission GCS Eyes	1 (1–2)	1 (1–2)	0.275
Admission GCS Verbal	1 (1–1)	1 (1–2)	0.246
Admission Pupil Response (% Bilaterally Reactive)	50%	67.20%	0.087
Marshall CT Grade	5 (3–5)	4.5 (3–5)	0.928
Rotterdam CT Grade	4.5 (4–5.25)	4 (4–5.25)	0.982
Helsinki CT Score	6 (4–9)	6 (5–9)	0.992
Stockholm CT Score	3.4 (2.9–3.91)	3.05 (2.4–3.9)	0.260
GOSE (6 Months)	6 (1–7)	5 (1–7)	0.445
Number Alive (GOSE 2–8) at 6 Months	65.40%	58.60%	0.594
Number Dead (GOSE 1) at 6 Months	34.60%	41.40%	0.594
Number Favorable (GOSE 5–8) at 6 Months	63.50%	55.20%	0.491
Number Unfavorable (GOSE 1–4) at 6 Months	36.50%	44.80%	0.491
Number with Hypoxia Episode	25%	39.10%	0.138
Number with Hypotension Episode	6.70%	12.50%	0.427
Number with Traumatic SAH	98.30%	95.30%	0.658
Number with Epidural Hematoma	10%	10.90%	1.000
Admission Hemoglobin	132 (119–146)	136 (113–148)	0.856
Admission Serum Glucose	8.2 (7–11.5)	8.2 (7.1–11)	0.815
Length of Hospital Stay	31 (10.2–53.8)	15 (7–32)	**0.032**
Length of ICU Stay	11 (6–16.5)	7 (3.5–13.5)	**0.017**
Mean MAP (mmHg)	84.5 (80.6–88.5)	83.8 (79–89.5)	0.677
Mean ICP (mmHg)	10.4 (6.59–13.1)	8.23 (5.19–12.2)	0.379
Mean Hourly Dose of ICP > 20 mmHg	5.79 (0.0561–13.8)	2.43 (0.0514–10.8)	0.338
Mean Hourly Dose of ICP > 22 mmHg	3.08 (0.00789–8.67)	0.933 (0–5.86)	0.274
Mean CPP (mmHg)	74.4 (71.2–77.9)	73.5 (69.8–80.6)	0.603
% Time CPP < 60 mmHg	4.36 (1.11–7.85)	3.4 (1.09–9.66)	0.755
% Time CPP > 70 mmHg	64.9 (51.4–74.6)	63.9 (43.7–78.7)	0.778
Mean PRx	0.152 (0.0316–0.277)	0.164 (0.0567–0.281)	0.729
% Time PRx > 0	65.3 (52.7–80.1)	68.6 (54.4–82.1)	0.575
% Time PRx > 0.25	39.7 (27.7–57.2)	42.2 (28.4–57.3)	0.648
% Time PRx > 0.35	30.7 (18.9–45.6)	31.7 (20–46.1)	0.688
Mean PAx	0.0325 (−0.109–0.147)	−0.0352 (−0.127–0.106)	0.481
% Time PAx > 0	54.2 (34.5–67.7)	45.5 (35–65.4)	0.589
% Time PAx > 0.20	33.8 (15.7–46.9)	24.7 (16.3–43.1)	0.444
% Time PAx > 0.25	28.8 (12.4–42.7)	21.3 (12.8–36.8)	0.401
Mean RAC	−0.205 (−0.439–−0.0487)	−0.248 (−0.379–−0.0327)	0.927
% Time RAC > 0	27.2 (13.3–43.8)	22.2 (15.1–44.4)	0.927

Bolded *p*-values are those reaching statistical significance, *p* < 0.05. CPP = cerebral perfusion pressure, CT = computed tomography, GCS = Glasgow Coma Scale, ICP = intracranial pressure, ICU = intensive care unit, IQR = interquartile range, MAP = mean arterial pressure, mmHg = millimeters of mercury, PAx = pulse amplitude index, PRx = pressure reactivity index, SAH = subarachnoid hemorrhage, RAC = correlation (R) between slow waves of AMP (A) and CPP (C).

## Appendix L

**Table A9:** Mann–Whitney U/chi-square testing for iICP (RAC > 0) identified vs. not identified.

Variable	Identified Median (IQR)	Not Identified Median (IQR)	*p*-Value
Age (years)	43 (32–59)	40 (25–53.5)	0.093
Sex (% Male)	79.60%	85.30%	0.556
Admission GCS Total	7 (5–8.25)	6 (4–8)	**0.035**
Admission GCS Motor	4 (3–5)	4 (2–5)	0.079
Admission GCS Eyes	1 (1–2.25)	1 (1–2)	0.095
Admission GCS Verbal	1 (1–2)	1 (1–1)	0.257
Admission Pupil Response (% Bilaterally Reactive)	61.20%	57.30%	0.911
Marshall CT Grade	5 (4–5)	4 (3–5)	**0.006**
Rotterdam CT Grade	5 (4–6)	4 (3–5)	**<0.001**
Helsinki CT Score	8 (5–9)	5 (4–9)	**0.018**
Stockholm CT Score	3.31 (2.5–3.95)	3.2 (2.5–3.8)	0.437
GOSE (6 Months)	5 (1–7)	6 (1–7)	0.371
Number Alive (GOSE 2–8) at 6 Months	53.30%	67.70%	0.185
Number Dead (GOSE 1) at 6 Months	46.70%	32.30%	0.185
Number Favorable (GOSE 5–8) at 6 Months	51.10%	64.60%	0.223
Number Unfavorable (GOSE 1–4) at 6 Months	48.90%	35.40%	0.223
Number with Hypoxia Episode	38.80%	28%	0.290
Number with Hypotension Episode	6.10%	12%	0.440
Number with Traumatic SAH	98%	96%	0.933
Number with Epidural Hematoma	10.20%	10.70%	1.000
Admission Hemoglobin	136 (122–150)	133 (113–146)	0.259
Admission Serum Glucose	8.3 (7.15–11)	8.2 (7.1–11.2)	0.915
Length of Hospital Stay	17 (8–42)	23 (10–41)	0.449
Length of ICU Stay	7 (4–13.8)	9 (4–15)	0.424
Mean MAP (mmHg)	81.3 (77.9–86.7)	85.5 (81.6–90.5)	**0.018**
Mean ICP (mmHg)	9.13 (5.01–12.4)	10 (7.05–12.8)	0.492
Mean Hourly Dose of ICP > 20 mmHg	1.21 (0–16.9)	4.18 (0.205–10.9)	0.367
Mean Hourly Dose of ICP > 22 mmHg	0.832 (0–7.25)	2.19 (0.0183–8.25)	0.356
Mean CPP (mmHg)	73.6 (69.1–76.7)	74.6 (71.1–80.5)	0.229
% Time CPP < 60 mmHg	4.73 (1.16–9.26)	3.76 (0.787–7.65)	0.421
% Time CPP > 70 mmHg	57.7 (41.3–74)	67.5 (51–82.3)	0.138
Mean PRx	0.184 (0.0641–0.289)	0.154 (0.0122–0.272)	0.268
% Time PRx > 0	69.4 (57–81.6)	63.9 (49.9–82.1)	0.180
% Time PRx > 0.25	43.6 (32.3–57.3)	39.9 (23.1–56.2)	0.202
% Time PRx > 0.35	31.9 (25.1–46.3)	29.3 (17.1–42.1)	0.239
Mean PAx	0.0483 (−0.0875–0.151)	−0.0467 (−0.14–0.0809)	**0.045**
% Time PAx > 0	59.2 (41.5–67.3)	43.4 (33–63.4)	0.052
% Time PAx > 0.20	36.5 (22.7–46.4)	22.7 (14.1–38.4)	**0.021**
% Time PAx > 0.25	30.8 (17.1–42.3)	18.9 (10.7–32.8)	**0.017**
Mean RAC	−0.124 (−0.279–0.00293)	−0.319 (−0.441–−0.124)	**<0.001**
% Time RAC > 0	36.1 (23.8–50.7)	19.6 (10.3–34.5)	**<0.001**

Bolded *p*-values are those reaching statistical significance, *p* < 0.05. CPP = cerebral perfusion pressure, CT = computed tomography, GCS = Glasgow Coma Scale, ICP = intracranial pressure, ICU = intensive care unit, IQR = interquartile range, MAP = mean arterial pressure, mmHg = millimeters of mercury, PAx = pulse amplitude index, PRx = pressure reactivity index, SAH = subarachnoid hemorrhage, RAC = correlation (R) between slow waves of AMP (A) and CPP (C).

## Appendix N

**Table A10:** Univariate models of iICP.ci and iICP.5-25 for Alive/Dead and Favorable/Unfavorable at 6 months.

**Alive/Dead Outcome Groups**				
**Model**	**AUC (95% CI)**	**AIC**	***p*** **-Value**	**Nagelkerke’s R^2^**
Mean Hourly Dose of ICP > 20 mmHg	0.614 (0.508–0.716)	138.3	**0.0217**	0.140
Mean Hourly Dose of ICP > 22 mmHg	0.623 (0.513–0.728)	139.4	**0.0146**	0.128
Mean Hourly Dose of ICP > iICP (PRx > 0)	0.660 (0.508–0.813)	68.6	**0.0293**	0.085
Mean Hourly Dose of ICP > iICP.ci (PRx > 0)	0.665 (0.485–0.829)	60.3	**0.0365**	0.076
Mean Hourly Dose of ICP > iICP.5-25 (PRx > 0)	0.762 (0.574–0.917)	44.3	**0.0042**	0.273
Mean Hourly Dose of ICP > iICP (PRx > 0.25)	0.530 (0.391–0.661)	102.8	0.3339	0.002
Mean Hourly Dose of ICP > iICP.ci (PRx > 0.25)	0.524 (0.370–0.684)	79.3	0.3842	0.002
Mean Hourly Dose of ICP > iICP.5-25 (PRx > 0.25)	0.541 (0.317–0.617)	79.1	0.3049	0.023
Mean Hourly Dose of ICP > iICP (PRx > 0.35)	0.503 (0.364–0.631)	108.7	0.4838	0.002
Mean Hourly Dose of ICP > iICP.ci (PRx > 0.35)	0.533 (0.375–0.691)	76.5	0.3444	0.001
Mean Hourly Dose of ICP > iICP.5-25 (PRx > 0.35)	0.510 (0.328–0.646)	81.1	0.4514	0.006
Mean Hourly Dose of ICP > iICP (PAx > 0)	0.533 (0.373–0.677)	89.2	0.3309	0.036
Mean Hourly Dose of ICP > iICP.ci (PAx > 0)	0.537 (0.274–0.649)	65.6	0.3441	0.030
Mean Hourly Dose of ICP > iICP.5-25 (PAx > 0)	0.552 (0.281–0.625)	69.2	0.2756	0.028
Mean Hourly Dose of ICP > iICP (PAx > 0.20)	0.481 (0.320–0.646)	77.4	0.5940	0.039
Mean Hourly Dose of ICP > iICP.ci (PAx > 0.20)	0.496 (0.278–0.718)	46.6	0.5220	0.027
Mean Hourly Dose of ICP > iICP.5-25 (PAx > 0.20)	0.473 (0.311–0.742)	46.5	0.6073	0.068
Mean Hourly Dose of ICP > iICP (PAx > 0.25)	0.624 (0.457–0.783)	66.2	0.0738	0.123
Mean Hourly Dose of ICP > iICP.ci (PAx > 0.25)	0.621 (0.379–0.856)	34.2	0.1694	0.166
Mean Hourly Dose of ICP > iICP.5-25 (PAx > 0.25)	0.561 (0.300–0.783)	37.5	0.3103	0.141
Mean Hourly Dose of ICP > iICP (RAC > 0)	0.554 (0.373–0.726)	63.7	0.2751	0.071
Mean Hourly Dose of ICP > iICP.ci (RAC > 0)	0.508 (0.280–0.758)	35.3	0.4880	0.033
Mean Hourly Dose of ICP > iICP.5-25 (RAC > 0)	0.665 (0.451–0.848)	43.3	0.0653	0.093
**Favorable/Unfavorable Outcome Groups**				
**Model**	**AUC (95% CI)**	**AIC**	***p*** **-Value**	**Nagelkerke’s R^2^**
Mean Hourly Dose of ICP > 20 mmHg	0.610 (0.500–0.719)	142.3	**0.0246**	0.123
Mean Hourly Dose of ICP > 22 mmHg	0.605 (0.490–0.711)	143.4	**0.0291**	0.111
Mean Hourly Dose of ICP > iICP (PRx > 0)	0.661 (0.492–0.808)	68.8	**0.0262**	0.134
Mean Hourly Dose of ICP > iICP.ci (PRx > 0)	0.663 (0.478–0.829)	60.8	**0.0337**	0.131
Mean Hourly Dose of ICP > iICP.5-25 (PRx > 0)	0.707 (0.499–0.880)	47.4	**0.0182**	0.224
Mean Hourly Dose of ICP > iICP (PRx > 0.25)	0.512 (0.353–0.628)	103.5	0.4361	0.001
Mean Hourly Dose of ICP > iICP.ci (PRx > 0.25)	0.557 (0.405–0.707)	79.6	0.2378	0.005
Mean Hourly Dose of ICP > iICP.5-25 (PRx > 0.25)	0.517 (0.333–0.634)	79.5	0.4173	0.017
Mean Hourly Dose of ICP > iICP (PRx > 0.35)	0.486 (0.384–0.642)	109.5	0.5859	0.001
Mean Hourly Dose of ICP > iICP.ci (PRx > 0.35)	0.569 (0.399–0.728)	76.9	0.1996	0.004
Mean Hourly Dose of ICP > iICP.5-25 (PRx > 0.35)	0.510 (0.338–0.636)	81.1	0.4514	0.006
Mean Hourly Dose of ICP > iICP (PAx > 0)	0.506 (0.351–0.665)	90.9	0.4721	0.027
Mean Hourly Dose of ICP > iICP.ci (PAx > 0)	0.492 (0.330–0.686)	67.4	0.5404	0.018
Mean Hourly Dose of ICP > iICP.5-25 (PAx > 0)	0.552 (0.273–0.620)	69.2	0.2756	0.028
Mean Hourly Dose of ICP > iICP (PAx > 0.20)	0.452 (0.384–0.719)	78.7	0.7291	0.032
Mean Hourly Dose of ICP > iICP.ci (PAx > 0.20)	0.496 (0.274–0.710)	46.6	0.5220	0.027
Mean Hourly Dose of ICP > iICP.5-25 (PAx > 0.20)	0.473 (0.308–0.731)	46.5	0.6073	0.068
Mean Hourly Dose of ICP > iICP (PAx > 0.25)	0.584 (0.403–0.748)	67.9	0.1631	0.109
Mean Hourly Dose of ICP > iICP.ci (PAx > 0.25)	0.621 (0.353–0.856)	34.2	0.1694	0.166
Mean Hourly Dose of ICP > iICP.5-25 (PAx > 0.25)	0.561 (0.306–0.800)	37.5	0.3103	0.141
Mean Hourly Dose of ICP > iICP (RAC > 0)	0.516 (0.338–0.694)	64.2	0.4331	0.062
Mean Hourly Dose of ICP > iICP.ci (RAC > 0)	0.508 (0.258–0.750)	35.3	0.4880	0.033
Mean Hourly Dose of ICP > iICP.5-25 (RAC > 0)	0.665 (0.464–0.853)	43.3	0.0653	0.093

Alive (GOSE 2–8), Dead (GOSE 1), Favorable (GOSE 5–8), Unfavorable (GOSE 1–4). Bolded *p*-values are those reaching statistical significance, *p* < 0.05. AIC = Akaike information criterion, AUC = area under the curve, CPP = cerebral perfusion pressure, CI = confidence interval, GOSE = Glasgow Outcome Score—Extended, ICP = intracranial pressure, iICP = individualized intracranial pressure threshold, iICP.ci = individualized intracranial pressure threshold (with CI maximum of 0.2), iICP.5-25 = individualized intracranial pressure threshold (with range limit of 5–25), mmHg = millimeters of mercury, PAx = pulse amplitude index, PRx = pressure reactivity index, RAC = correlation (R) between slow waves of AMP (A) and CPP (C).

## Appendix O

**Table A11:** Multivariable models of iICP.ci and iICP.5-25 for Alive/Dead and Favorable/Unfavorable at 6 months.

**Alive/Dead Outcome Groups**					
**Model**	**AUC (95% CI)**	**AIC**	***p*** **-Value**	**Nagelkerke’s R^2^**	**Δ Nagelkerke’s R^2^**
IMPACT Core	0.793 (0.698–0.879)	124.2	**<0.0001**	0.344	-
+ Mean Hourly Dose of ICP > 20 mmHg	0.855 (0.773–0.926)	107.9	**<0.0001**	0.500	0.156
+ Mean Hourly Dose of ICP > 22 mmHg	0.852 (0.768–0.925)	109.5	**<0.0001**	0.487	0.143
+ Mean Hourly Dose of ICP > iICP (PRx > 0)	0.914 (0.817–0.984)	49.3	**<0.0001**	0.607	0.263
+ Mean Hourly Dose of ICP > iICP.ci (PRx > 0)	0.898 (0.783–0.981)	46.5	**<0.0001**	0.566	0.222
+ Mean Hourly Dose of ICP > iICP.5-25 (PRx > 0)	0.988 (0.946–1.000)	22.3	**<0.0001**	0.878	0.534
+ Mean Hourly Dose of ICP > iICP (PRx > 0.25)	0.801 (0.684–0.904)	88.3	**<0.0001**	0.358	0.014
+ Mean Hourly Dose of ICP > iICP.ci (PRx > 0.25)	0.762 (0.617–0.894)	74.4	**0.0004**	0.282	−0.062
+ Mean Hourly Dose of ICP > iICP.5-25 (PRx > 0.25)	0.869 (0.771–0.951)	60.0	**<0.0001**	0.534	0.190
+ Mean Hourly Dose of ICP > iICP (PRx > 0.35)	0.788 (0.672–0.883)	93.8	**<0.0001**	0.346	0.002
+ Mean Hourly Dose of ICP > iICP.ci (PRx > 0.35)	0.781 (0.635–0.909)	70.5	**0.0002**	0.313	−0.031
+ Mean Hourly Dose of ICP > iICP.5-25 (PRx > 0.35)	0.828 (0.705–0.931)	67.4	**<0.0001**	0.432	0.088
+ Mean Hourly Dose of ICP > iICP (PAx > 0)	0.708 (0.570–0.827)	86.3	**0.0024**	0.237	−0.107
+ Mean Hourly Dose of ICP > iICP.ci (PAx > 0)	0.673 (0.504–0.824)	68.4	**0.0256**	0.166	−0.178
+ Mean Hourly Dose of ICP > iICP.5-25 (PAx > 0)	0.763 (0.635–0.884)	68.7	**0.0008**	0.233	−0.111
+ Mean Hourly Dose of ICP > iICP (PAx > 0.20)	0.698 (0.545–0.827)	77.2	**0.0063**	0.217	−0.127
+ Mean Hourly Dose of ICP > iICP.ci (PAx > 0.20)	0.746 (0.544–0.909)	49.7	**0.0099**	0.211	−0.133
+ Mean Hourly Dose of ICP > iICP.5-25 (PAx > 0.20)	0.712 (0.523–0.869)	50.8	**0.0217**	0.205	−0.139
+ Mean Hourly Dose of ICP > iICP (PAx > 0.25)	0.742 (0.585–0.869)	66.8	**0.0019**	0.290	−0.054
+ Mean Hourly Dose of ICP > iICP.ci (PAx > 0.25)	0.712 (0.490–0.895)	40.4	**0.0423**	0.245	−0.099
+ Mean Hourly Dose of ICP > iICP.5-25 (PAx > 0.25)	0.711 (0.494–0.894)	42.1	**0.0359**	0.280	−0.064
+ Mean Hourly Dose of ICP > iICP (RAC > 0)	0.756 (0.601–0.893)	64.5	**0.0014**	0.259	−0.085
+ Mean Hourly Dose of ICP > iICP.ci (RAC > 0)	0.674 (0.424–0.879)	41.2	**0.0847**	0.142	−0.202
+ Mean Hourly Dose of ICP > iICP.5-25 (RAC > 0)	0.750 (0.558–0.915)	47.3	**0.0097**	0.249	−0.095
**Favorable/Unfavorable Outcome Groups**					
**Model**	**AUC (95% CI)**	**AIC**	***p*** **-Value**	**Nagelkerke’s R^2^**	**Δ Nagelkerke’s R^2^**
IMPACT Core	0.753 (0.653–0.845)	135.1	**<0.0001**	0.262	-
+ Mean Hourly Dose of ICP > 20 mmHg	0.801 (0.706–0.884)	123.1	**<0.0001**	0.392	0.130
+ Mean Hourly Dose of ICP > 22 mmHg	0.799 (0.701–0.889)	124.7	**<0.0001**	0.378	0.116
+ Mean Hourly Dose of ICP > iICP (PRx > 0)	0.871 (0.752–0.965)	56.6	**<0.0001**	0.523	0.261
+ Mean Hourly Dose of ICP > iICP.ci (PRx > 0)	0.857 (0.722–0.959)	52.9	**<0.0001**	0.486	0.224
+ Mean Hourly Dose of ICP > iICP.5-25 (PRx > 0)	0.903 (0.746–1.000)	39.1	**<0.0001**	0.616	0.354
+ Mean Hourly Dose of ICP > iICP (PRx > 0.25)	0.780 (0.662–0.889)	91.3	**<0.0001**	0.325	0.063
+ Mean Hourly Dose of ICP > iICP.ci (PRx > 0.25)	0.755 (0.613–0.883)	76.4	**0.0005**	0.251	−0.011
+ Mean Hourly Dose of ICP > iICP.5-25 (PRx > 0.25)	0.848 (0.735–0.940)	63.2	**<0.0001**	0.488	0.226
+ Mean Hourly Dose of ICP > iICP (PRx > 0.35)	0.770 (0.647–0.872)	96.7	**<0.0001**	0.317	0.055
+ Mean Hourly Dose of ICP > iICP.ci (PRx > 0.35)	0.779 (0.638–0.901)	70.1	**0.0002**	0.328	0.066
+ Mean Hourly Dose of ICP > iICP.5-25 (PRx > 0.35)	0.828 (0.703–0.935)	67.4	**<0.0001**	0.432	0.170
+ Mean Hourly Dose of ICP > iICP (PAx > 0)	0.705 (0.565–0.824)	89.1	**0.0024**	0.208	−0.054
+ Mean Hourly Dose of ICP > iICP.ci (PAx > 0)	0.693 (0.519–0.838)	70.0	**0.0130**	0.160	−0.102
+ Mean Hourly Dose of ICP > iICP.5-25 (PAx > 0)	0.763 (0.633–0.880)	68.7	**0.0008**	0.233	−0.029
+ Mean Hourly Dose of ICP > iICP (PAx > 0.20)	0.684 (0.542–0.814)	80.0	**0.0097**	0.179	−0.083
+ Mean Hourly Dose of ICP > iICP.ci (PAx > 0.20)	0.746 (0.556–0.909)	49.7	**0.0099**	0.211	−0.051
+ Mean Hourly Dose of ICP > iICP.5-25 (PAx > 0.20)	0.712 (0.527–0.877)	50.8	**0.0217**	0.205	−0.057
+ Mean Hourly Dose of ICP > iICP (PAx > 0.25)	0.726 (0.574–0.858)	69.8	**0.0033**	0.250	−0.012
+ Mean Hourly Dose of ICP > iICP.ci (PAx > 0.25)	0.712 (0.490–0.895)	40.4	**0.0423**	0.245	−0.017
+ Mean Hourly Dose of ICP > iICP.5-25 (PAx > 0.25)	0.711 (0.489–0.894)	42.1	**0.0359**	0.280	0.018
+ Mean Hourly Dose of ICP > iICP (RAC > 0)	0.739 (0.575–0.874)	65.4	**0.0027**	0.241	−0.021
+ Mean Hourly Dose of ICP > iICP.ci (RAC > 0)	0.674 (0.417–0.879)	41.2	**0.0847**	0.142	−0.120
+ Mean Hourly Dose of ICP > iICP.5-25 (RAC > 0)	0.750 (0.558–0.911)	47.3	**0.0097**	0.249	−0.013

IMPACT Core model consists of age, admission Glasgow Coma Scale–motor score, and admission pupillary response. Alive (GOSE 2–8), Dead (GOSE 1), Favorable (GOSE 5–8), Unfavorable (GOSE 1–4). Bolded *p*-values are those reaching statistical significance, *p* < 0.05. AIC = Akaike information criterion, AUC = area under the curve, CPP = cerebral perfusion pressure, CI = confidence interval, GOSE = Glasgow Outcome Score—Extended, ICP = intracranial pressure, iICP = individualized intracranial pressure threshold, iICP.ci = individualized intracranial pressure threshold (with CI maximum of 0.2), iICP.5-25 = individualized intracranial pressure threshold (with range limit of 5–25), IMPACT = International Mission for Prognosis and Analysis of Clinical Trials, mmHg = millimeters of mercury, PAx = pulse amplitude index, PRx = pressure reactivity index, RAC = correlation (R) between slow waves of AMP (A) and CPP (C).
